# Intrinsic polyunsaturated fatty acid synthesis capacity dictates ferroptosis sensitivity under restricted arachidonic acid availability

**DOI:** 10.1038/s41420-026-03240-6

**Published:** 2026-07-11

**Authors:** Min Wook Kim, Seo Young Jang, Ji-Yoon Lee, Jong Woo Kim, Woosung Jung, Yeon-Mi You, Jooeun Kim, Kyoung-Jin Oh, Won Kon Kim, Baek-Soo Han, Kwang-Hee Bae, Mi-Young Kim, Geum-Sook Hwang, Eun-Woo Lee

**Affiliations:** 1 Department of Biological Science, Korea Advanced Institute of Science and Technology (KAIST), Daejeon, Republic of Korea; 2 Metabolic Regulation Research Center, Korea Research Institute of Bioscience and Biotechnology (KRIBB), Daejeon, Republic of Korea; 3 Integrated Metabolomics Research Group, Metropolitan Seoul Center, Korea Basic Science Institute (KBSI), Seoul, Korea; 4 Department of Functional Genomics, University of Science and Technology (UST), Daejeon, Republic of Korea; 5 Biodefense Research Center, Korea Research Institute of Bioscience and Biotechnology (KRIBB), Daejeon, Republic of Korea; 6 College of Pharmacy, Chung-Ang University, Seoul, Republic of Korea; 7 School of Pharmacy, Sungkyunkwan University, Suwon, Republic of Korea

**Keywords:** Lipidomics, Cell death, Non-small-cell lung cancer, Cancer microenvironment

## Abstract

Ferroptosis is an iron-dependent type of regulated cell death driven by lipid peroxidation, in which polyunsaturated fatty acids (PUFAs) in membrane phospholipids serve as key substrates. Here, we identify PUFA biosynthetic capacity as a key determinant of ferroptosis sensitivity under arachidonic acid (AA)-limited conditions. Lipidomic and stable isotope-tracing analyses in human lung adenocarcinoma cell lines revealed that H1299 cells that harbor wild-type KEAP1 and are sensitive to ferroptosis have unexpectedly impaired PUFA biosynthesis and consequently low PUFA levels. Nevertheless, ferroptosis sensitivity in H1299 cells is maintained under normal culture conditions owing to an exogenous supply of AA. Culturing cells in the B27 supplement, which provides only essential fatty acids such as linoleic acid, in the presence of 1% dialyzed FBS reduced AA-containing phospholipids and rendered H1299 cells resistant to ferroptosis. In contrast, A549 cells with a KEAP1 mutation retained ferroptosis sensitivity under B27 conditions, enabled by their endogenous PUFA-synthesizing capacity. Strikingly, pharmacological inhibition of *FADS2* or deletion of *ELOVL5* in A549 cells phenocopied H1299 cells, conferring resistance under B27-AO conditions that was reversed by AA supplementation. These findings demonstrate that ferroptosis susceptibility is dictated by intrinsic PUFA biosynthetic capacity under AA-limited conditions resembling physiological lipid availability. Therefore, intrinsic PUFA biosynthetic capacity should be considered in ferroptosis-based cancer therapies.

## Introduction

Ferroptosis is driven by iron-catalyzed peroxidation of membrane phospholipids [1, 2], which predominantly targets polyunsaturated fatty acids (PUFAs) at the sn-2 position and leads to the accumulation of oxidized phospholipids, ultimately compromising membrane integrity [3, 4]. In particular, phosphatidylethanolamine (PE) and phosphatidylcholine (PC) species esterified with arachidonic acid (AA; 20:4n-6) or adrenic acid (AdA; 22:4n-6) serve as pivotal substrates for lipid peroxidation, thereby driving ferroptosis [5–7]. These highly oxidizable PUFA-containing phospholipids are specifically incorporated into membranes by enzymes such as acyl-CoA synthetase long-chain family member 4 (ACSL4) [8, 9] and lysophosphatidylcholine acyltransferase 3 (LPCAT3) [10, 11], a process that determines cellular susceptibility to ferroptosis [12–14].

Specifically, PUFA biosynthesis proceeds through the sequential desaturation and elongation of linoleic acid (LA; 18:2n-6) to generate AA [12, 15, 16]. This pathway begins with the fatty acid desaturase 2 (FADS2)-mediated desaturation of LA to γ-linolenic acid (GLA; 18:3n-6), followed by elongation to dihomo-γ-linolenic acid (DGLA; 20:3n-6) by enzymes of the elongation of very long-chain fatty acids (ELOVL) family, and is completed by a final desaturation step catalyzed by FADS1 to produce AA [12]. We previously reported that mesenchymal-type gastric cancer (GC) cells are highly sensitive to ferroptosis due to their ability to synthesize AA from LA, whereas intestinal-type GC cells are resistant to ferroptosis owing to disrupted PUFA biosynthesis caused by the loss of *FADS1* or *ELOVL5*, underscoring the pivotal role of PUFA synthesis in determining ferroptosis sensitivity [17]. Moreover, FADS2 has emerged as a rate-limiting regulator of ferroptosis, as its role in PUFA desaturation governs cellular susceptibility to ferroptotic death across diverse contexts [18–21]. Notably, cancer cells frequently undergo lipid metabolic reprogramming, including alterations in PUFA biosynthetic pathways, which contribute to malignancy, therapy resistance, and metastatic progression. However, these metabolic changes can simultaneously generate vulnerabilities to ferroptosis [16, 21–25].

In addition to intrinsic metabolic remodeling, extracellular lipid availability, particularly the uptake of extracellular PUFAs, has been shown to shape ferroptosis responses in cancer cells by altering the membrane phospholipid composition [25–27]. Importantly, fetal bovine serum (FBS), which is widely used in cell culture, contains little LA but appreciable amounts of AA, whereas human serum and plasma are enriched in LA and contain only minimal AA [28–31]. This discrepancy indicates that de novo PUFA synthesis from LA is more critical for regulating ferroptosis in vivo than under standard culture conditions [26]. Therefore, elucidating how cancer cells reprogram lipid metabolism to coordinate PUFA synthesis and uptake may provide critical insights into the mechanisms governing ferroptosis, particularly under physiologically AA-limited conditions in the tumor microenvironment.

In lung adenocarcinoma, the interplay between the nuclear factor erythroid 2-related factor 2 (NRF2)/Kelch-like ECH-associated protein 1 (KEAP1) pathway and the ferroptosis suppressor protein 1 (FSP1)-coenzyme Q (CoQ) antioxidant axis modulates ferroptosis sensitivity [32–36]. However, how this regulatory network interacts with intrinsic PUFA synthesis and extracellular lipid availability remains unclear. In this study, we hypothesized that intrinsic differences in PUFA biosynthetic capacity dictate ferroptosis sensitivity under AA-limited conditions. To test this hypothesis, we utilized two widely used human lung adenocarcinoma cell lines, A549 and H1299, which differ in key genetic alterations, including KRAS, p53, STK11, and KEAP1. While Ras and p53 also influence ferroptosis in a context-dependent manner, the KEAP1-NRF2-FSP1 axis appears to play a dominant role, as targeting FSP1 markedly sensitizes lung adenocarcinoma cells to ferroptosis [32, 35]. Interestingly, H1299 cells, which harbor wild-type KEAP1, exhibit impaired PUFA biosynthesis, whereas A549 cells, which carry KEAP1 mutations, retain active PUFA biosynthetic capacity. Despite defective PUFA biosynthesis, H1299 cells remained sensitive to ferroptosis under standard culture conditions, consistent with previous studies, indicating that exogenous lipids can compensate for their biosynthetic deficiency. However, under B27-supplemented, AA-limited conditions, H1299 cells became resistant to ferroptosis. In contrast, A549 cells retain ferroptosis sensitivity under both culture conditions, consistent with their intrinsic capacity for PUFA synthesis. Together, these findings highlight that intrinsic PUFA biosynthetic capacity becomes a decisive determinant of ferroptosis sensitivity under conditions in which exogenous pro-ferroptotic lipids are limited.

## Results

### Basal PUFA abundance does not predict ferroptosis sensitivity

**Fig. 1:**
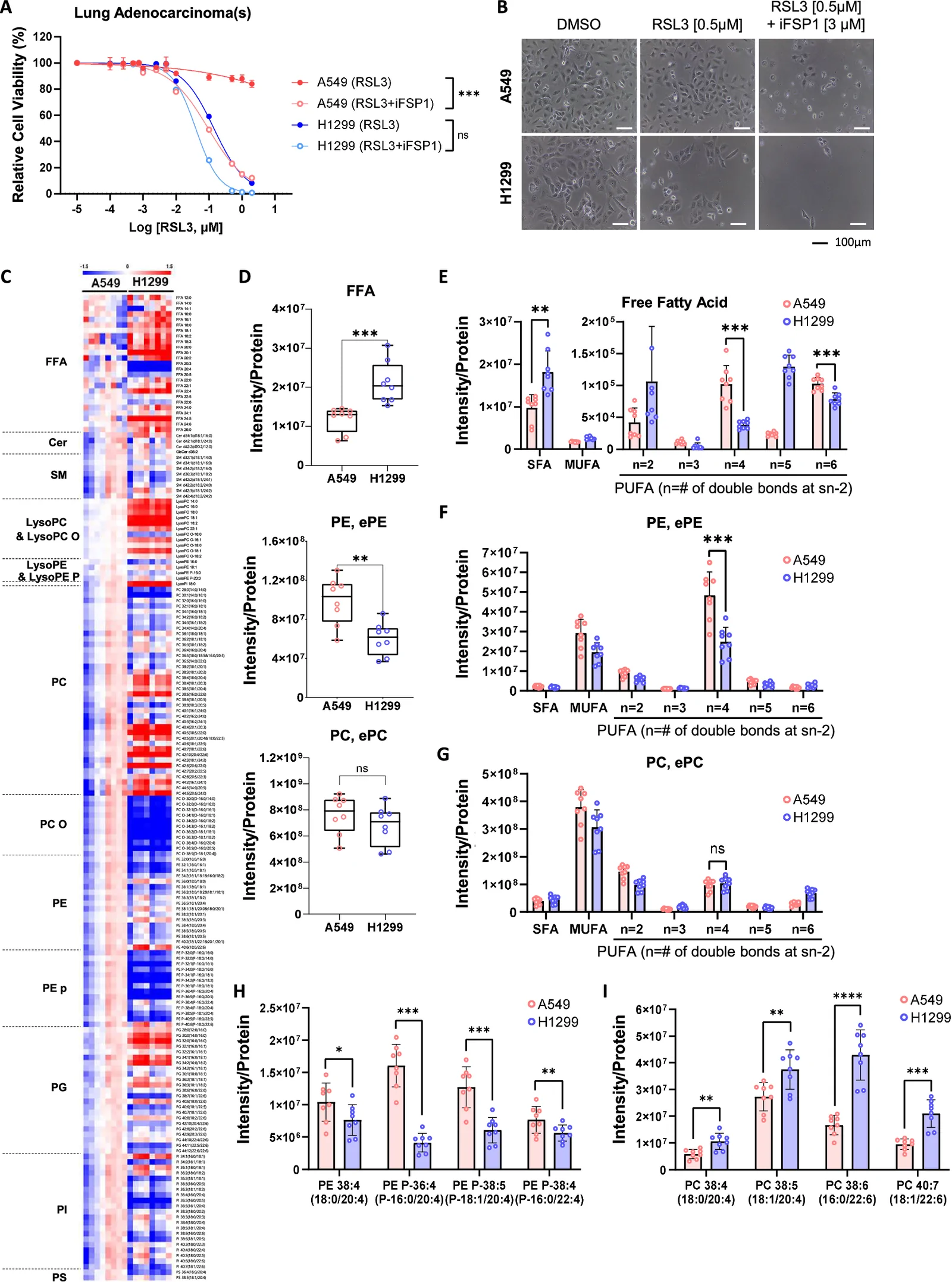
Lipidomic heterogeneity defines divergent ferroptosis phenotypes in A549 and H1299 cells. **A** Relative viability of A549 and H1299 cells treated with RSL3 for 16 h at the indicated concentrations with or without iFSP1 (3 μM). Data represent mean ± SD from *n* = 3 independent experiments. Statistical significance was determined using the Mann–Whitney U test (*P < 0.05; **P < 0.01; ***P < 0.001; ns, not significant). **B** Representative morphology of A549 and H1299 cells under the same conditions as in (**A**). Scale bar, 100 μm. **C** Global lipidomic profiles analyzed by LC–MS/MS and normalized to protein (*n* = 8 independent experiments). **D** Total amounts of free fatty acids (FFAs), phosphatidylethanolamine (PE and ether-linked PE; ePE), and phosphatidylcholine (PC and ether-linked PC; ePC), normalized to protein (*n* = 8 independent experiments). Saturation-dependent distributions of FFA (**E**), PE/ePE (**F**), and PC/ePC (**G**) species (*n* = 8 independent experiments). **H** Representative PUFA-containing PE species (PE 38:4, PE-P 36:4, PE-P 38:5, and PE-P 38:4) that were more abundant in A549 cells than in H1299 cells, shown as detected intensity normalized to protein (*n* = 8 independent experiments). **I** Representative PUFA-containing PC species (PC 38:4, PC 38:5, PC 38:6, and PC 40:7) that were more abundant in H1299 cells than in A549 cells, shown as detected intensity normalized to protein (*n* = 8 independent experiments).

In non-small cell lung cancer (NSCLC), ferroptosis sensitivity has been closely linked to antioxidant defense pathways, particularly the KEAP1-NRF2 axis and the FSP1 pathway [32, 33, 35, 37]. However, the extent to which basal lipid composition modulates ferroptosis sensitivity in NSCLC remains poorly defined. Here, we compared two NSCLC cell lines with well-defined ferroptosis sensitivities, A549 and H1299. A549 cells harbor a KEAP1 mutation that constitutively activates NRF2 and exhibit high FSP1 expression, both of which contribute to their ferroptosis-resistant phenotype [32, 33, 37]. In contrast, H1299 cells express wild-type KEAP1 and are highly sensitive to ferroptosis. As expected, A549 cells were resistant to the GPX4 inhibitor RSL3 but became sensitive upon co-treatment with the FSP1 inhibitor (iFSP1), whereas H1299 cells showed strong sensitivity to RSL3 alone, with little additional effect of iFSP1 (Fig. 1A, B).

To assess whether intrinsic differences in lipid composition contribute to these phenotypes, we analyzed the lipidomic profiles of A549 and H1299 cells. Lipidomic data revealed distinct lipid compositions between the two cell lines (Fig. 1C). Despite these distinctions, the overall abundances of phosphatidylcholine (PC), phosphatidylinositol (PI), phosphatidylglycerol (PG), phosphatidylserine (PS), and sphingomyelin (SM) were comparable between the two cell lines (Fig. 1D and Supplementary Fig. S1A). Notably, the total free fatty acid (FFA) pool was elevated, whereas PE species were generally reduced in H1299 cells compared with A549 cells (Fig. 1D). Based on this observation, we next examined saturation-dependent distributions across major lipid subclasses, including FFA, PE, and PC (Fig. 1E–G). Although total amounts of saturated (SFA), monounsaturated (MUFA), and PUFA were comparable between the two cell lines (Supplementary Fig. S1B), H1299 cells displayed elevated levels of free SFAs, MUFAs, and a subset of PUFAs (Fig. 1D, E). In contrast, the levels of PUFAs containing three or four double bonds, including DGLA and AA, were markedly reduced in H1299 cells (Supplementary Fig. S1C). Together, these findings suggest a redistribution of lipid species rather than a global difference in total PUFA abundance between the two cell lines.

We next focused on PUFA-containing phospholipids that function as key substrates for ferroptosis. Unexpectedly, PE and ether-linked PE (ePE) species harboring PUFAs with four double bonds were markedly decreased in ferroptosis-sensitive H1299 cells compared with A549 cells (Fig. 1F, H). In contrast, the overall profiles of PUFA-containing PC and ether-linked PC (ePC) species were largely comparable between the two cell lines (Fig. 1G, I). Notably, although PE and ePE species containing PUFAs with four double bonds were markedly reduced in H1299 cells (Fig. 1F), these lipids remained detectable (Fig. 1H). Although the overall saturation profile of PC species showed no major differences between the two cell lines (Fig. 1G), H1299 cells exhibited increased levels of AA- or docosahexaenoic acid (DHA, 22:6)-containing PC species (Fig. 1I). These results indicate that although H1299 cells contain reduced levels of PE-PUFAs, they retain a detectable PE-PUFA pool and exhibit enrichment of selected AA- or DHA-containing PC species. Thus, the basal abundance of PUFA-containing lipids alone does not directly predict ferroptosis sensitivity under standard culture conditions.

### Selective downregulation of FADS2 impairs PUFA biosynthesis in H1299 cells

**Fig. 2:**
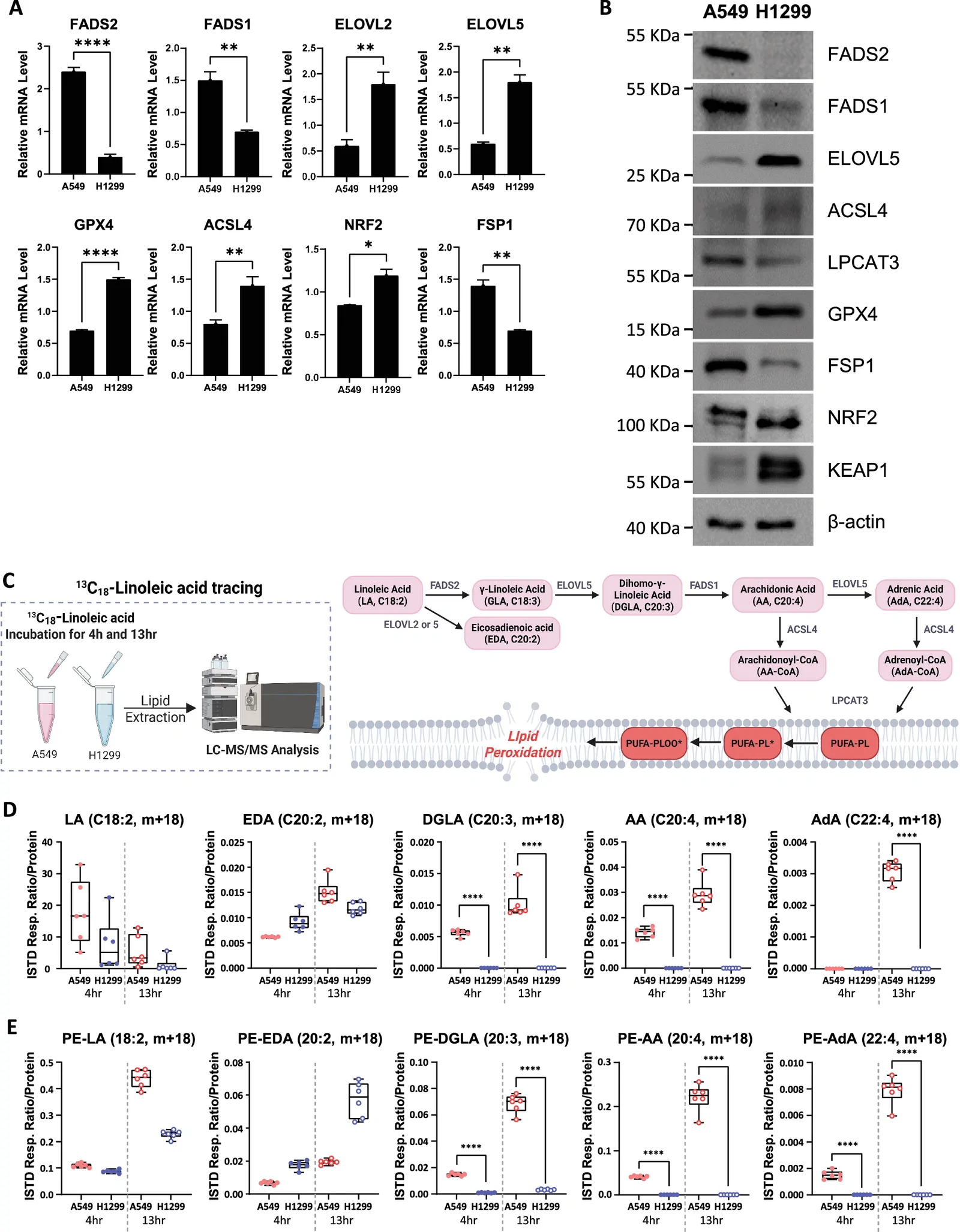
Intrinsic PUFA biosynthetic capacity shapes ferroptosis sensitivity. **A** Relative mRNA expression of PUFA synthesis–related genes normalized to β-actin (*n* = 3 independent experiments). Data represent mean ± SD. Statistical significance was determined using the Mann–Whitney U test. **B** Immunoblot analysis of PUFA pathway proteins with β-actin as a loading control. For ELOVL5 detection, samples were not boiled. **C** Schematic of the stable ^13^${{\rm{C}}}_{18}$-labeled linoleic acid (LA) tracing assay. Incorporation of ^13^${{\rm{C}}}_{18}$-labeled LA into free fatty acids (**D**) and PE species (**E**) after 4 h and 13 h, normalized to protein (*n* = 6 independent experiments). Data represent mean ± SD from *n* = 6 independent experiments. Statistical significance was determined by one-way ANOVA followed by Tukey’s multiple-comparison test (*P < 0.05; **P < 0.01; ***P < 0.001; ****P < 0.0001; ns, not significant).

Given that DGLA and AA levels were markedly lower in H1299 cells (Supplementary Fig. S1C), we examined the expression of key enzymes involved in the ω-6 PUFA biosynthetic pathway. qRT–PCR analysis revealed marked downregulation of *FADS2* and a more modest reduction in *FADS1* expression in H1299 cells compared with A549 cells (Fig. 2A), which was consistent at the protein level as confirmed by immunoblotting (Fig. 2B). Interestingly, *ELOVL2/5* expression was elevated in H1299 cells compared with A549 cells (Fig. 2A, B). Consistent with previous reports [12, 33, 37], A549 cells carrying KEAP1 mutations displayed NRF2 activation and FSP1 overexpression, whereas H1299 cells exhibited relatively higher levels of GPX4 expression, as confirmed by both qRT–PCR and immunoblotting (Fig. 2A, B).

Because the opposing expression patterns of *FADS2* and *ELOVL2/5* made it difficult to infer overall PUFA biosynthetic activity, we next performed stable isotope tracing using ^13^C18-labeled LA and analyzed downstream metabolites using LC‒MS/MS (Fig. 2C). Both A549 and H1299 cells showed comparable levels of ^13^C18-labeled LA incorporation at 4 h and similar residual amounts at 13 h in the medium (Supplementary Fig. S2), suggesting that their rates of LA acquisition and utilization were largely comparable. After 4 h and 13 h of labeling, A549 cells produced labeled DGLA and AA, consistent with active metabolic flux through the desaturation and elongation pathway. In contrast, H1299 cells accumulated labeled LA and eicosadienoic acid (EDA; C20:2), but failed to generate detectable DGLA, AA, or AdA at either time point, indicating a block at the desaturation step (Fig. 2C, D).

When we assessed incorporation of newly synthesized PUFAs into membrane phospholipids, A549 cells exhibited robust incorporation of labeled LA and AA into PE species, whereas H1299 cells incorporated labeled LA and EDA but showed negligible incorporation of DGLA or AA (Fig. 2E). Taken together, these results demonstrate that selective downregulation of *FADS2* in H1299 cells impairs PUFA desaturation, thereby reducing the levels of downstream products such as AA and AdA in membrane phospholipids.

### PUFA synthesis defects drive ferroptosis resistance under exogenous AA-limited conditions

As H1299 cells are unable to synthesize downstream PUFAs from LA yet still retain ferroptosis sensitivity under standard culture conditions, we hypothesized that they may rely on exogenous AA present in FBS. Because standard FBS contains relatively low LA but appreciable AA, it does not accurately mimic the physiological composition of human serum and plasma, which are rich in LA and poor in AA [28–31]. To model an exogenous AA-limited environment, we utilized B27 supplementation, which provides only essential fatty acids such as LA and α-linolenic acid (ALA; C18:3n-3), thereby forcing cells to rely more directly on the endogenous PUFA biosynthetic pathway.

However, recent studies have highlighted that B27 supplements contain antioxidants, including vitamin A, vitamin E, selenium, N-acetylcysteine, catalase, and superoxide dismutase, which can inadvertently suppress ferroptosis [38–40]. To exclude confounding effects, we used an antioxidant-free formulation (B27-AO), although it still contains vitamin A, and compared it with dialyzed FBS (dFBS). As expected, culturing cells in dFBS enhanced ferroptosis sensitivity relative to standard FBS (Supplementary Fig. S3A). Notably, A549 cells became sensitive under dFBS even without iFSP1 co-treatment, indicating that reducing exogenous antioxidants can bypass FSP1-dependent protection (Supplementary Fig. S3B). When we attempted to adapt cells to B27-AO alone, neither H1299 nor A549 cells survived, indicating that a minimal level of serum-derived factors is required to support cell viability during subculture (Supplementary Fig. S3C). Thus, both cell lines could be maintained in B27-AO supplemented with 1% dFBS (hereafter referred to as B27-AO conditions) (Supplementary Fig. S3C), which supplies the minimal serum-derived factors while restricting exogenous AA availability.

To directly analyze fatty acid composition, we first examined the lipid composition of the stock solutions, including FBS, dFBS, B27-AO, and pooled human plasma standard reference material (SRM). As expected, B27-AO contained two essential fatty acids, including LA (C18:2n-6) and ALA (C18:3n-3). Notably, while human plasma also contained abundant levels of these two essential fatty acids, FBS and dFBS contained low levels of LA and ALA (Supplementary Fig. S3D). We next examined fatty acid profiles under each culture condition. Notably, B27-AO supplemented with 1% dFBS contained comparable amounts of LA and ALA to human plasma, whereas 10% dFBS contained markedly lower levels of these fatty acids. In contrast, 10% dFBS contained high levels of AA (C20:4), whereas AA levels were limited in both B27-AO/1% dFBS and human plasma, with B27-AO conditions exhibiting the lowest AA abundance among the groups (Supplementary Fig. S3E). B27-AO conditions also contained additional polyunsaturated fatty acids (PUFAs), likely derived from the 1% dFBS component, as confirmed by direct analysis (Supplementary Fig. S3D). Finally, principal component analysis (PCA) demonstrated that the overall lipidomic profile of B27-AO conditions clustered more closely with human plasma than with conventional serum-based conditions such as 10% dFBS (Supplementary Fig. S3F). These results provide direct experimental evidence supporting our rationale for using B27-AO supplemented with 1% dFBS as an AA-limited condition that more closely mimics a physiological lipid environment. Therefore, we adopted these conditions for subsequent experiments.

**Fig. 3:**
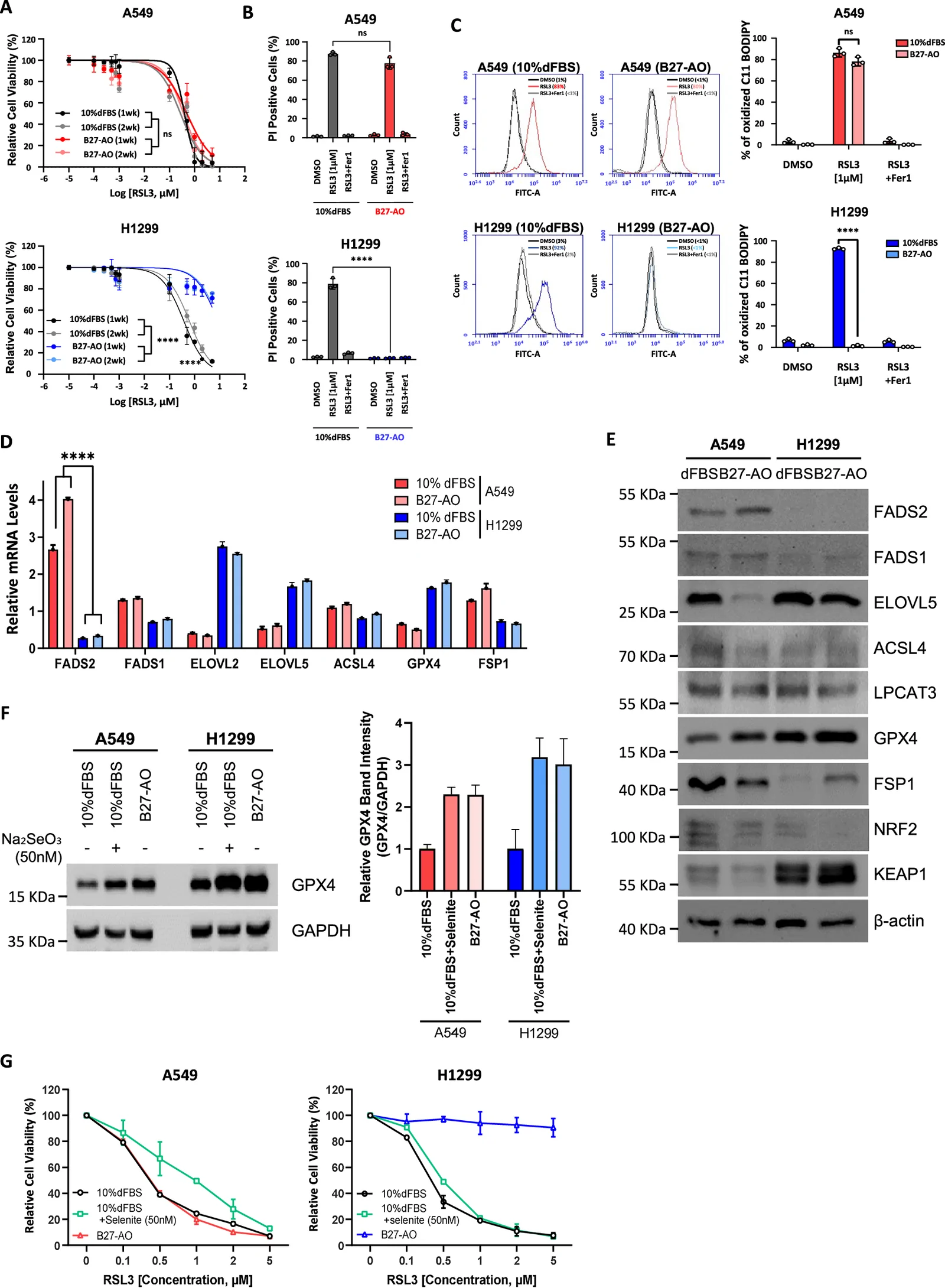
PUFA synthesis capacity determines ferroptosis outcomes under arachidonic acid–restricted conditions. **A** Relative viability of A549 and H1299 cells treated with RSL3 (1 μM) for 20 h after 1- or 2-week adaptation to 10% dialyzed FBS (dFBS) or B27 supplement without antioxidants in the presence of 1% dFBS (B27-AO) (*n* = 3 independent experiments). **B** Propidium iodide (PI) uptake analysis by flow cytometry in A549 and H1299 cells treated with DMSO, RSL3 (1 μM), or RSL3 (1 μM) co-treated with Fer-1 (2 μM) (*n* = 3 independent experiments). **C** Lipid peroxidation analysis by C11-BODIPY staining in A549 and H1299 cells cultured in dFBS or B27-AO/1% dFBS and treated with RSL3 (1 μM) or RSL3 with Fer-1 (2 μM) for 30 min (*n* = 3 independent experiments). **D** Relative mRNA expression levels of *FADS2, FADS1, ELOVL2, ELOVL5, ACSL4, GPX4*, and *FSP1* in A549 and H1299 cells cultured in dFBS or B27-AO/1% dFBS (*n* = 3 independent experiments). All mRNA expression levels were normalized to β-actin mRNA levels. **E** Immunoblot analysis of the indicated proteins under the same conditions. β-actin was used as a loading control. **F** Immunoblot analysis of GPX4 expression in A549 and H1299 cells cultured in dFBS with or without sodium selenite (50 nM), compared with B27-AO/1% dFBS. GAPDH was used as a loading control. Quantification of GPX4 band intensity normalized to GAPDH is shown (*n* = 3 independent experiments, mean ± SD), with a representative blot. **G** Relative viability of A549 and H1299 cells cultured in dFBS with or without sodium selenite (50 nM) and treated with increasing concentrations of RSL3 (0–5 μM) for 20 h (*n* = 3 independent experiments). For **A–D,** **G**, data represent mean ± SD from *n* = 3 independent experiments. Statistical significance was determined using non-parametric tests: the Mann–Whitney U test for two-group comparisons and the Kruskal–Wallis test followed by Dunn’s multiple-comparison test for multiple-group comparisons, as appropriate (*P < 0.05; **P < 0.01; ***P < 0.001; ****P < 0.0001; ns, not significant).

To evaluate ferroptosis sensitivity under AA-restricted conditions, we treated A549 and H1299 cells with RSL3 after one or two weeks of culture in either 10% dFBS or B27-AO. A549 cells showed comparable viability across both conditions and underwent robust ferroptotic cell death, consistent with their ability to synthesize AA through de novo PUFA biosynthesis, indicating that exogenous AA is not required for their ferroptosis sensitivity (Fig. 3A). In contrast, H1299 cells became resistant to ferroptosis under B27-AO conditions, but remained sensitive under 10% dFBS conditions (Fig. 3A). To further validate these observations, we quantified PI uptake (Fig. 3B) and C11-BODIPY oxidation (Fig. 3C) as indicators of necrotic cell death and lipid peroxidation, respectively. In A549 cells, RSL3 treatment induced strong necrotic cell death under both dFBS and B27-AO conditions, whereas H1299 cells displayed minimal PI uptake under B27-AO (Fig. 3B). Both A549 and H1299 cells exhibited robust C11-BODIPY oxidation in dFBS, whereas H1299 cells maintained markedly reduced lipid oxidation under B27-AO (Fig. 3C). Taken together, the PI uptake and lipid peroxidation data demonstrate that ferroptotic responses are preserved in A549 cells regardless of exogenous AA availability, whereas H1299 cells show greatly diminished ferroptotic activity specifically under AA-limited conditions.

To investigate pathways beyond PUFA synthesis that might influence ferroptosis sensitivity under B27-AO, we examined the expression of key enzymes involved in PUFA biosynthesis at both the transcript and protein levels (Fig. 3D, E). Among these enzymes, *FADS2* expression showed the most pronounced difference between the two cell lines, with consistently higher levels in A549 cells and lower levels in H1299 cells under both dFBS and B27-AO conditions. In contrast, ELOVL5 protein levels were reduced under B27-AO conditions in both cell lines, but this change did not correlate with the observed differences in ferroptosis sensitivity (Fig. 3E).

Notably, GPX4 levels increased under B27-AO in both cell lines, likely due to the presence of sodium selenite in B27-AO (Fig. 3E) [38]. This finding suggested that elevated GPX4 could contribute to the ferroptosis resistance observed in H1299 cells. To test this hypothesis, we supplemented cells cultured in 10% dFBS with sodium selenite, which increased GPX4 protein levels to levels comparable to those observed under B27-AO (Fig. 3F). Although selenium treatment increased GPX4 protein levels, it did not alter the cellular response to RSL3-induced ferroptosis, as both A549 and H1299 cells remained comparably sensitive (Fig. 3G). These findings indicate that increased GPX4 expression alone cannot explain the selective resistance of H1299 cells under AA-limited conditions and support the conclusion that impaired PUFA synthesis is the primary driver of ferroptosis resistance in this context.

### Lipidomic remodeling under limited exogenous AA availability reveals selective loss of ferroptosis-relevant phospholipids

**Fig. 4:**
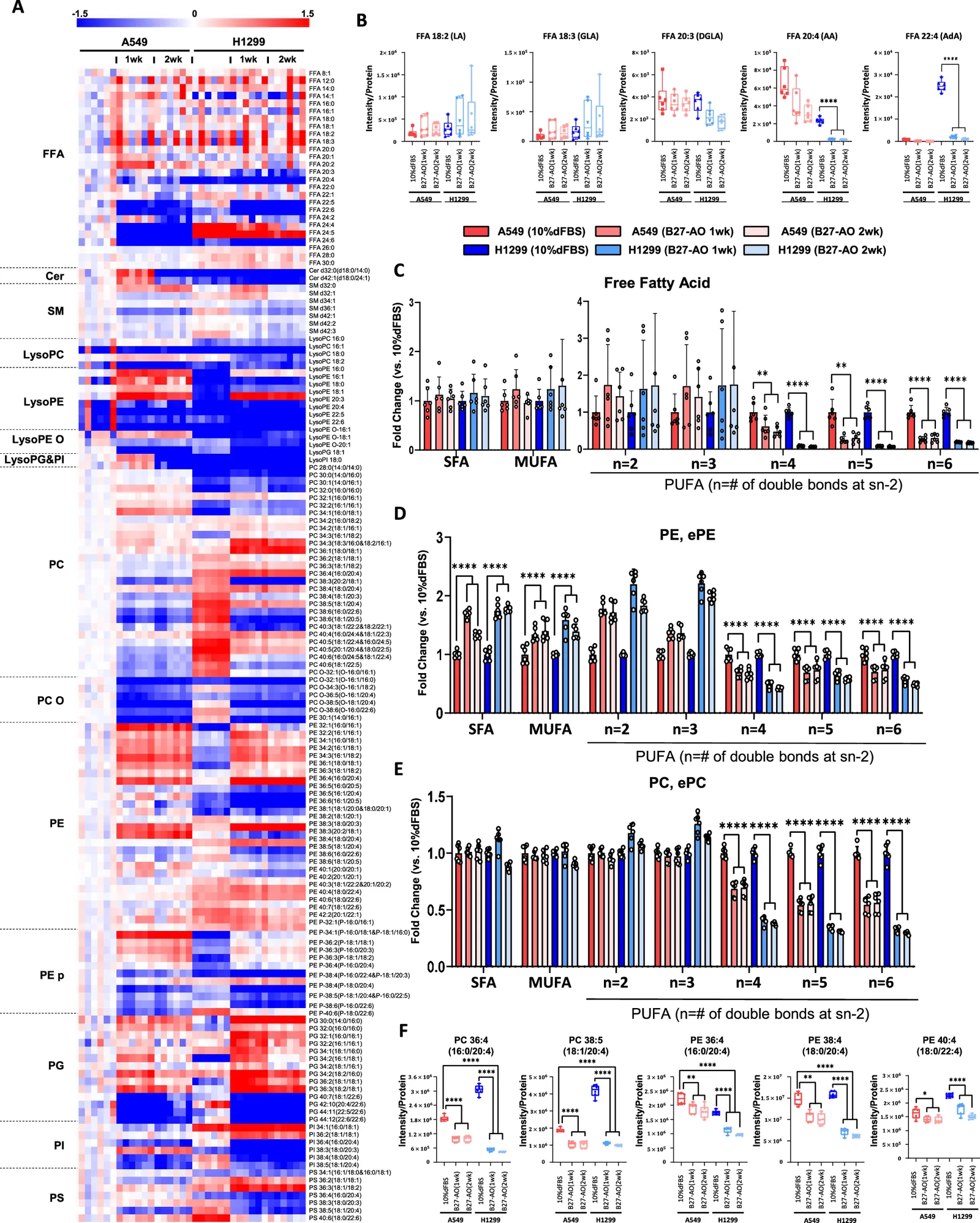
Lipidomic remodeling under arachidonic acid–restricted conditions reveals selective loss of PUFA-containing phospholipids. **A** Heatmap of untargeted lipidomic profiles in A549 and H1299 cells cultured in 10% dialyzed FBS (dFBS) or adapted to B27 supplement without antioxidants in the presence of 1% dFBS (B27-AO) for 1 or 2 weeks (*n* = 6 independent experiments). **B** Relative abundance of free fatty acids in the PUFA biosynthetic pathway, including linoleic acid (LA), γ-linolenic acid (GLA), dihomo-γ-linolenic acid (DGLA), arachidonic acid (AA), and adrenic acid (AdA), in A549 and H1299 cells cultured in dFBS or B27-AO/1% dFBS (*n* = 6 independent experiments). **C** Fold change in free fatty acid (FFA) species categorized by double-bond number in A549 and H1299 cells cultured in dFBS or adapted to B27-AO/1% dFBS for 1 or 2 weeks, calculated relative to the 10% dFBS condition within each cell line (baseline = 1) (*n* = 6 independent experiments). **D** Fold change in phosphatidylethanolamine and ether-linked PE (PE/ePE) species categorized by double-bond number in A549 and H1299 cells cultured in dFBS or adapted to B27-AO/1% dFBS for 1 or 2 weeks, calculated relative to the 10% dFBS condition within each cell line (baseline = 1) (*n* = 6 independent experiments). **E** Fold change in phosphatidylcholine and ether-linked PC (PC/ePC) species categorized by double-bond number in A549 and H1299 cells cultured in dFBS or adapted to B27-AO/1% dFBS for 1 or 2 weeks, calculated relative to the 10% dFBS condition within each cell line (baseline = 1) (*n* = 6 independent experiments). **F** Representative PUFA-containing phospholipid species selected based on VIP scores, including PC 36:4 (16:0/20:4), PC 38:5 (18:1/20:4), PE 36:4 (16:0/20:4), PE 38:4 (18:0/20:4), and PE 40:4 (18:0/22:4), showing pronounced depletion in H1299 cells upon adaptation to B27-AO/1% dFBS. All lipid abundances were normalized to protein (*n* = 6 independent experiments). Data represent mean ± SD from *n* = 6 independent experiments. Statistical significance was determined by one-way ANOVA followed by Tukey’s multiple-comparison test (*P < 0.05; **P < 0.01; ***P < 0.001; ****P < 0.0001; ns, not significant).

We next profiled global lipidomic changes in A549 and H1299 cells cultured in B27-AO to assess how exogenous AA limitation alters lipid composition relevant to ferroptosis (Fig. 4A). Partial least squares discriminant analysis (PLS-DA) confirmed clear separation between dFBS-grown and B27-adapted cells (Supplementary Fig. S4A). Building on these global lipidomic shifts, we first examined FFAs in the PUFA biosynthetic pathway (Fig. 4B). LA and GLA levels remained comparable between the two cell lines and were largely unaffected by B27-AO, whereas DGLA was stably maintained in A549 cells but markedly decreased in H1299 cells specifically under B27-AO (Fig. 4B). Strikingly, AA levels in H1299 cells, already lower than in A549 cells, were drastically reduced upon B27-AO, consistent with defective PUFA synthesis (Fig. 4B). When we analyzed the unsaturation profiles of all FFA species across conditions, PUFAs with four to six double bonds were selectively depleted in H1299 cells under B27-AO, whereas SFAs, MUFAs and PUFAs with two or three double bonds remained largely unchanged (Fig. 4C and Supplementary Fig. S4C).

Given that specific phospholipid species critically modulate ferroptosis sensitivity, we next examined PE and PC species, the major phospholipids involved in lipid peroxidation during ferroptosis [8] under B27-AO conditions. PE and ether-linked PE (PE/ePE) species enriched in highly unsaturated PUFAs with three to six double bonds showed the most pronounced changes, exhibiting a substantial reduction in H1299 cells upon B27-AO adaptation (Fig. 4D and Supplementary Fig. S4C). Although A549 cells also showed a reduction in PUFA-containing PE species, the magnitude of reduction was considerably smaller, indicating that PE-PUFA depletion is more pronounced in H1299 cells. In parallel, MUFA-containing PE species increased in both cell lines following B27-AO adaptation, with a more prominent shift in H1299 cells. We next analyzed PUFA-containing PC species categorized by the number of double bonds (Fig. 4E and Supplementary Fig. S4C). Under basal (dFBS) conditions, H1299 cells showed higher levels of PC-PUFAs than A549 cells. However, B27-AO adaptation caused a dramatic reduction in these species in H1299 cells, decreasing them to levels that were comparable to, or in some cases even lower than, those observed in A549 cells. Thus, both PE- and PC-based PUFA pools underwent marked depletion in H1299 cells under AA-restricted conditions.

Meanwhile, A549 cells also exhibited a moderate decrease in PUFA-containing PE and PC species under B27-AO conditions (Fig. 4D, E). The remaining PUFA-rich phospholipid pool appeared sufficient to sustain lipid peroxidation and ferroptotic cell death, consistent with their preserved ferroptosis sensitivity. To identify lipid species that most strongly contributed to the observed compositional shifts, we calculated variable importance in projection (VIP) scores. This analysis highlighted PUFA-containing phospholipids, such as PC 36:4 (16:0/20:4), PC 38:5 (18:1/20:4), PE 36:4 (16:0/20:4), PE 38:4 (18:0/20:4), and PE 40:4 (18:0/22:4), key discriminating lipids contributing to the separation of B27-AO-adapted H1299 cells from the other groups. Collectively, these lipidomic changes, characterized by depletion of oxidizable PE-PUFA species and a compensatory enrichment of MUFA-rich phospholipids, delineate a remodeled membrane landscape that underlies ferroptosis resistance in H1299 cells under AA-restricted conditions.

### Exogenous AA supplementation rescues ferroptosis under AA-limited conditions

**Fig. 5:**
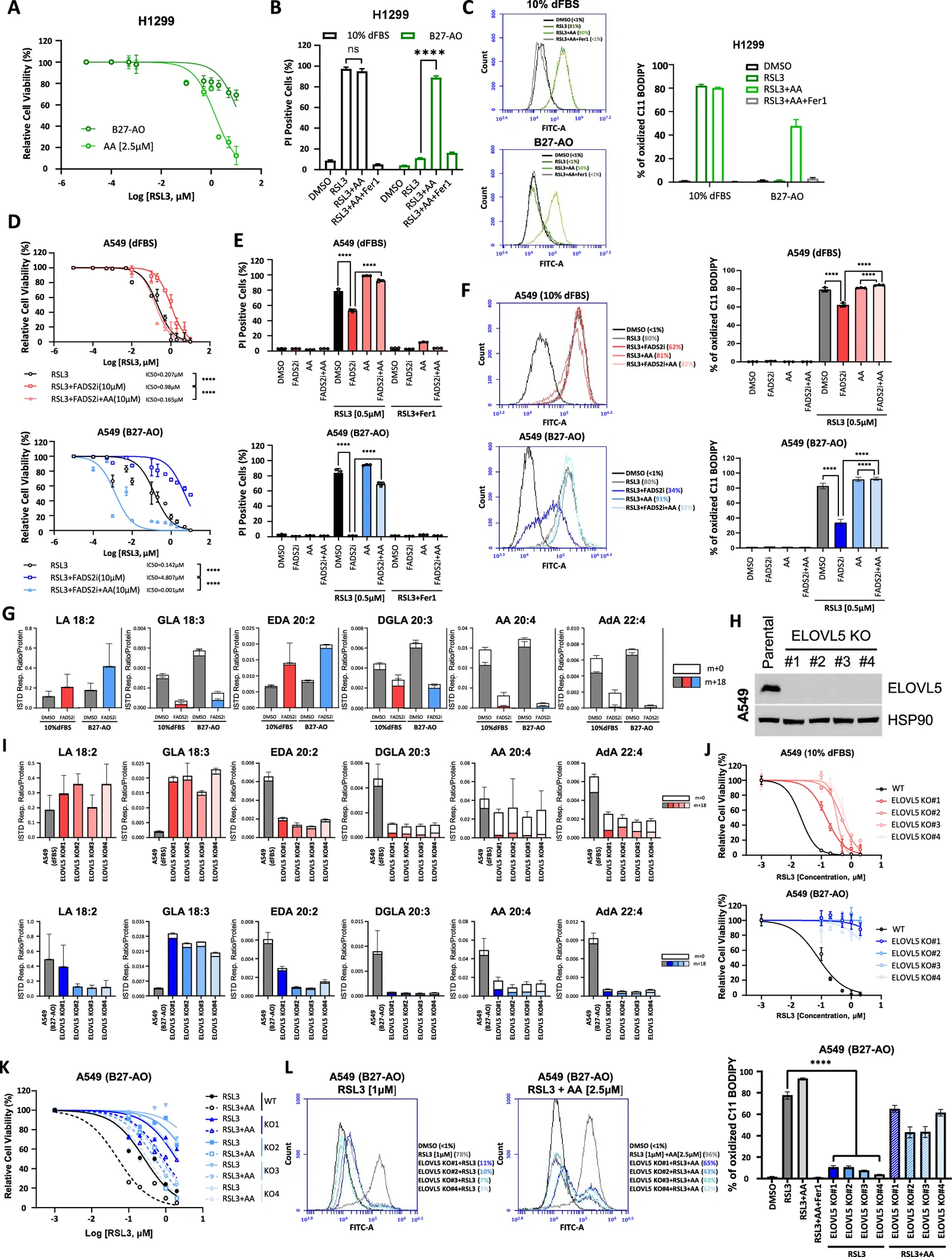
Exogenous AA supplementation and loss of endogenous PUFA biosynthesis bidirectionally regulate ferroptosis sensitivity under AA-limited conditions. **A** Relative viability of H1299 cells cultured under the indicated conditions, pretreated with arachidonic acid (AA, 2.5 μM) for 24 h, and subsequently treated with RSL3 for 20 h (*n* = 3 independent experiments). **B** Propidium iodide (PI) uptake analysis in H1299 cells pretreated with arachidonic acid (AA, 2.5 μM) for 24 h and subsequently treated with DMSO, RSL3 (1 μM), or RSL3 in combination with ferrostatin-1 (Fer-1, 2 μM) for 20 h (*n* = 3 independent experiments). **C** Lipid peroxidation analysis by C11-BODIPY staining in H1299 cells under the indicated conditions. Left, representative histograms. Right, quantification of lipid peroxidation measured 1 h after RSL3 treatment (*n* = 3 independent experiments). **D** Relative viability of A549 cells cultured in dFBS or B27-AO/1% dFBS and treated with a *FADS2* inhibitor (10 μM) under the indicated conditions, with or without AA supplementation (2.5 μM, 24 h pretreatment), followed by RSL3 treatment for 20 h (*n* = 3 independent experiments). **E** Propidium iodide (PI) uptake analysis in A549 cells cultured in dFBS or B27-AO/1% dFBS and treated with a *FADS2* inhibitor (10 μM) under the indicated conditions, with or without AA supplementation (2.5 μM, 24 h pretreatment), followed by RSL3 treatment (*n* = 3 independent experiments). **F** Lipid peroxidation analysis by C11-BODIPY staining in A549 cells cultured in dFBS or B27-AO/1% dFBS and treated with a *FADS2* inhibitor (10 μM) under the indicated conditions, with or without AA supplementation (2.5 μM, 24 h pretreatment). Lipid peroxidation was measured 1 h after RSL3 treatment (*n* = 3 independent experiments). **G** ^13^${{\rm{C}}}_{18}$-labeled LA tracing analysis in A549 cells cultured in dFBS or B27-AO/1% dFBS with or without *FADS2* inhibitor treatment (5 μM), showing incorporation into PUFA biosynthetic intermediates and downstream products under the indicated conditions (*n* = 6 independent experiments). **H** Immunoblot analysis of wild-type (WT) and *ELOVL5* knockout (KO) A549 cells. HSP90 was used as a loading control. For ELOVL5 detection, samples were not boiled. **I** ^13^${{\rm{C}}}_{18}$-labeled LA tracing analysis of WT and *ELOVL5* KO cells under the indicated conditions, showing loss of downstream PUFA biosynthetic flux following *ELOVL5* deletion (*n* = 6 independent experiments). **J** Relative viability of WT and *ELOVL5* KO A549 cells after 2 weeks of adaptation to dFBS or B27-AO/1% dFBS and subsequent treatment with RSL3 for 20 h (*n* = 3 independent experiments). **K** Relative viability of WT and *ELOVL5* KO A549 cells cultured in B27-AO/1% dFBS under the indicated conditions, with or without AA supplementation (2.5 μM, 24 h pretreatment), followed by RSL3 treatment for 20 h (*n* = 3 independent experiments). **L** Lipid peroxidation analysis by C11-BODIPY staining in WT and *ELOVL5* KO A549 cells under the indicated conditions, with or without AA supplementation (2.5 μM, 24 h pretreatment). Lipid peroxidation was measured 1 h after RSL3 treatment (*n* = 3 independent experiments). For panels **A**–**F** and **J**–**L**, data represent mean ± SD from *n* = 3 independent experiments. Statistical significance was determined using non-parametric tests: the Mann–Whitney U test for two-group comparisons and the Kruskal–Wallis test followed by Dunn’s multiple-comparison test for multiple-group comparisons, as appropriate. For **G, I**, data represent mean ± SD from *n* = 6 independent experiments. Statistical significance was determined by one-way ANOVA followed by Tukey’s multiple-comparison test. (*P < 0.05; **P < 0.01; ***P < 0.001; ****P < 0.0001; ns, not significant).

Because reduced PUFA-containing phospholipids in H1299 cells under B27-AO correlated with ferroptosis resistance, we tested whether exogenous AA supplementation could restore ferroptosis sensitivity. AA supplementation markedly increased RSL3-induced cell death and lipid peroxidation in H1299 cells under B27-AO, demonstrating that H1299 cells rely on external AA to support ferroptotic responses when endogenous PUFA biosynthetic capacity is impaired (Fig. 5A–C). In contrast, A549 cells remained ferroptosis-sensitive under B27-AO owing to their intact PUFA biosynthetic capacity, and exogenous AA produced only a modest additional effect (Supplementary Figs. S5A–C).

We next asked whether the loss of PUFA biosynthetic capacity in A549 cells could mimic the resistant phenotype observed in H1299 cells. Since A549 cells express *FADS2,* whereas H1299 cells show low *FADS2* expression, we employed a *FADS2* inhibitor, SC-26196. Treatment with the *FADS2* inhibitor increased resistance to RSL3-induced ferroptosis, and exogenous AA supplementation restored ferroptosis sensitivity in these cells (Fig. 5D–F). To directly test whether these phenotypic changes reflected impaired PUFA biosynthesis, we performed ^13^C_18_-labeled LA tracing and found that *FADS2* inhibition markedly suppressed PUFA biosynthesis, as shown by reduced incorporation into ^13^C-labeled (m + 18) GLA (C18:3), DGLA (C20:3), and AA (C20:4) under both dFBS and B27-AO conditions (Fig. 5G). In contrast, EDA (C20:2) accumulated upon *FADS2* inhibition, suggesting a metabolic shift toward LA elongation rather than desaturation (Fig. 5G). While AA synthesis from LA was markedly impaired upon *FADS2* inhibition, unlabeled AA (m + 0) was still maintained under dFBS conditions, likely due to uptake from the medium. In contrast, under B27-AO conditions, extracellular AA availability was restricted, resulting in markedly reduced m + 0 AA levels. These findings are consistent with the stronger suppression of ferroptosis by pharmacological inhibition of *FADS2* under B27-AO conditions, where cells cannot compensate for impaired endogenous AA synthesis through extracellular AA uptake (Fig. 5G).

We then asked whether genetic deletion of *ELOVL5*, another key enzyme in the PUFA synthesis pathway, in A549 cells could mimic pharmacological *FADS2* inhibition (Fig. 5H) [17]. Although ELOVL5 protein expression in A549 cells was lower than in H1299 cells under FBS conditions (Fig. 2B) and further decreased under B27-AO conditions (Fig. 3E), stable isotope-tracing analysis indicated that ELOVL5-dependent elongation activity was preserved, as evidenced by comparable conversion of LA (C18:2) to EDA (C20:2) under different conditions (Fig. 5G, I). Consistent with this, *ELOVL5* deficiency caused a marked defect in PUFA synthesis, as evidenced by reduced ^13^C-labeled (m + 18) EDA, DGLA, AA, and AdA under both dFBS and B27-AO culture conditions, suggesting that ELOVL5 is a major elongase required for arachidonic acid synthesis in these cells (Fig. 5I). Interestingly, *ELOVL5* deficiency resulted in accumulation of the FADS2 desaturation product GLA (C18:3), consistent with a metabolic bottleneck at the elongation step (Fig. 5H). In addition, *ELOVL5* deficiency markedly increased uptake of extracellular AA (m + 0) under dFBS conditions, suggesting a strong compensatory uptake of extracellular AA to maintain intracellular AA levels. However, under B27-AO conditions, where extracellular AA is limited, both m + 0 AA and total AA levels were markedly reduced in *ELOVL5* KO cells (Fig. 5I).

Compared with wild-type A549 cells, A549-*ELOVL5* knockout (KO) cells remained sensitive to RSL3 treatment in dFBS, although with slightly reduced responsiveness (Fig. 5J). However, adaptation to B27-AO rendered A549-*ELOVL5* KO cells strongly resistant to ferroptosis, phenocopying the resistance observed in H1299 cells under the same conditions (Fig. 5J). Importantly, supplementation with exogenous AA restored both lipid peroxidation and ferroptosis sensitivity in A549-*ELOVL5* KO cells, indicating that impaired endogenous PUFA synthesis is a major driver of ferroptosis resistance under AA-limited conditions (Fig. 5K, L).

Together, these findings establish AA availability as a central metabolic checkpoint for ferroptosis, positioning endogenous PUFA biosynthesis and exogenous PUFA supply as key regulators of cell fate under nutrient-limited conditions. Given that human serum and plasma contain abundant LA but are relatively poor in AA, reliance on the endogenous PUFA biosynthetic pathway is likely to be greater in vivo, suggesting that impaired biosynthetic capacity may represent a critical determinant of ferroptosis resistance under physiological conditions.

## Discussion

Our comparative lipidomic analysis of A549 and H1299 NSCLC cell lines revealed that ferroptosis susceptibility is not dictated by the overall abundance of PUFAs but rather by how oxidizable PUFAs are distributed across specific phospholipid classes [5, 8]. H1299 cells contained markedly lower levels of AA than A549 cells due to impaired PUFA biosynthesis (Supplementary Fig. S1C), yet retained a minimal pool of PE species containing PUFAs with four double bonds, including AA, which may account for their residual ferroptosis sensitivity (Supplementary Fig. S1D) [12, 17]. In addition, H1299 cells exhibited higher levels of AA- and DHA-containing PC species, the latter also reported as a potent ferroptosis-activating lipid [41]. In contrast, A549 cells contained abundant PUFA-PE species, which are typically considered pro-ferroptotic lipids, but remained resistant, likely because of their KEAP1 mutation and concurrently elevated FSP1 expression, both of which counteract lipid peroxidation [33, 34]. Together, these findings indicate that strong antioxidant defenses can override the intrinsic vulnerability created by peroxidation-prone phospholipids, emphasizing that ferroptosis sensitivity in these cells reflects the balance between lipid class composition and antioxidant capacity [42].

Building upon these insights, we further demonstrate that extracellular lipid availability critically shapes ferroptosis responses, particularly in cells with impaired PUFA biosynthesis. A549 cells, capable of de novo PUFA synthesis, preserved ferroptosis sensitivity regardless of lipid source. In contrast, H1299 cells became resistant under B27-AO conditions, in which AA availability is limited, consistent with reduced *FADS2* expression limiting conversion of LA into downstream PUFAs. Consistent with this notion, prolonged adaptation (1–2 weeks) to B27-AO media revealed pronounced lipidomic remodeling in H1299 cells, characterized by depletion of PUFA-containing phospholipid pools (Fig. 4C–E). This loss was accompanied by an increase in MUFA-enriched phospholipids, reflecting their inability to sustain oxidizable lipid pools under AA-restricted conditions.

In contrast, A549 cells preserved a PUFA-rich lipid composition even under prolonged restricted conditions, likely due to intact endogenous PUFA biosynthesis that maintains production of oxidizable lipids despite limited exogenous input. Notably, although ELOVL5 protein levels appeared reduced under B27-AO conditions, stable isotope-tracing analysis indicated continued downstream PUFA production, suggesting that the remaining ELOVL5 activity may be sufficient to sustain flux through the PUFA biosynthetic pathway and that protein abundance alone may not fully reflect functional enzymatic capacity. Therefore, pharmacological inhibition of *FADS2* or genetic deletion of *ELOVL5* in A549 cells phenocopied H1299 cells, maintaining ferroptosis sensitivity under dFBS conditions while becoming resistant under AA-limited conditions. Strikingly, exogenous AA supplementation restored ferroptosis sensitivity in H1299 cells, as well as in A549 cells with *FADS2* inhibition or *ELOVL5* deletion, confirming that impaired PUFA synthesis drives resistance under AA-restricted conditions. These results highlight lipid availability as a critical determinant of ferroptosis, and PUFA-deficient cells evade ferroptosis under AA-restricted conditions yet readily regain sensitivity when supplied with exogenous AA. Notably, although perturbation of both *FADS2* and *ELOVL5* results in similar defects in AA biosynthesis, targeting the desaturation and elongation steps can lead to distinct metabolic outcomes, as we observed that FADS2 inhibition promoted LA elongation to EDA while ELOVL5 deficiency enhanced LA desaturation to GLA. Therefore, the impact of targeting each step of PUFA biosynthesis on global lipidomic alterations warrants further investigation to develop more effective and safer strategies for ferroptosis-related diseases.

A critical hurdle in translating in vitro findings to in vivo relevance lies in the discrepancy between cell culture media and human plasma, which has been recognized as a major contributor to the frequent failure of drug candidates in clinical trials [43]. To overcome this limitation, human serum- and plasma–like media have been developed to better mimic physiological metabolite levels, including urea, glucose, glutamine, and antioxidants [43, 44]. However, the lipid composition of culture media supplemented with fetal bovine serum (FBS) has received comparatively little attention. Human serum and plasma are enriched in LA but contain little AA, whereas FBS provides relatively limited LA and appreciable amounts of AA [28–31]. This discrepancy may artificially shift cells toward reliance on AA uptake rather than synthesis. Although our study focuses on lipid availability as a key determinant of ferroptosis sensitivity, we cannot completely exclude the possibility that other components present in B27-AO or serum may also contribute to the observed differences between culture conditions. To better approximate the physiological context, we employed B27 lacking antioxidants supplemented with 1% dFBS. Although B27 is widely used in neuronal and organoid cultures, its antioxidant activity can suppress ferroptosis, necessitating careful consideration [38].

Accumulating evidence underscores the importance of AA in ferroptosis within the tumor microenvironment. A recent study reported comparable levels of AA in tumor interstitial fluid, suggesting that cancer cells may directly acquire AA from their surroundings [45, 46]. Moreover, CD${8}^{+}$ T cells require ACSL4-dependent AA metabolism to undergo ferroptosis, and CD36-mediated AA uptake has been shown to promote T-cell ferroptosis, thereby facilitating tumor growth [27]. These findings indicate that competition for AA among cancer cells, immune cells, and other stromal populations, together with their differential capacity for de novo PUFA synthesis, may critically shape ferroptosis sensitivity in vivo. In addition to free fatty acids, phospholipids and lipoproteins can also act as sources of oxidizable lipids [47–49], further underscoring the complexity of lipid exchange in the tumor microenvironment and its impact on ferroptosis sensitivity.

Together, our findings suggest an integrated model in which ferroptosis susceptibility in NSCLC cells is determined by the interplay between intrinsic PUFA biosynthetic capacity and the availability of exogenous lipids. Under physiological conditions where exogenous AA is limited, endogenous PUFA synthesis becomes essential for maintaining ferroptotic competence. Thus, the ferroptosis sensitivity observed under standard culture conditions should be carefully interpreted in the physiological context, as several cancer cells have often lost the ability to synthesize their own PUFAs. Understanding how PUFA metabolism and antioxidant mechanisms are coordinated across distinct cell types in the tumor microenvironment will be crucial for developing therapeutic strategies for NSCLC.

## Materials and methods

### Reagents

RSL3 (S8155) and Ferrostatin-1 (S7243) were purchased from Selleck Chemicals (USA). iFSP1 (HY-136057) was obtained from MedChemExpress (USA). C11 BODIPY 581/591 (D3861) was purchased from Thermo Fisher Scientific (USA). Arachidonic acid (AA; 90010) and the FADS2 inhibitor (SC-26196) were obtained from Cayman Chemical (USA), and ^13^C_18_- linoleic acid (^13^C_18_-LA; 605735) was obtained from Sigma‒Aldrich (USA). All the chemicals were stored at −20 °C until use.

### Lipidomic analysis of intracellular lipids using LC-MS/MS

Intracellular lipids were extracted by adding 600 μL of ice-cold methanol: water (80:20, v/v) containing 0.5% formic acid to cells cultured in 6-well plates. Tridecanoic acid (0.5 μM, FFA 13:0, Sigma–Aldrich, 91988) and a 100-fold diluted SPLASH Lipidomix (Avanti Polar Lipids, 330707), covering multiple lipid classes, were added to the extraction solution as internal standards. After washing with DPBS, cells were rapidly quenched and scraped, followed by centrifugation at 18,500 × *g* for 20 min at 4 °C to obtain protein-free lipid extracts. A total of 500 μL of the supernatant was transferred to a new microcentrifuge tube, followed by adding 200 μL of water and 800 μl of chloroform. The mixture was vortexed vigorously for 1 min and centrifuged at 13,800 × *g* for 20 min at 4 °C. The lower organic phase containing lipids was collected. The lipid extracts were dried under nitrogen gas and reconstituted in 400 μl of isopropanol/acetonitrile/water (50:25:25, v/v/v). Lipidomic analysis was performed using a Xevo G2-XS QTOF mass spectrometer coupled to an ACQUITY UPLC system (Waters) at the Korea Basic Science Institute (KBSI, WS005). Chromatographic separation was achieved using an ACQUITY UPLC CSH C18 column (2.1 × 100 mm, 1.7 μm; Waters) maintained at 55 °C. The mobile phases for negative ion mode consisted of (A) 10 mM ammonium acetate in water: acetonitrile (60:40, v/v) and (B) isopropanol: acetonitrile (90:10, v/v). The flow rate was set to 0.4 mL/min with the following gradient: 40–43% B (0–2 min), 43–50% B (2–2.1 min), 50–54% B (2.1–12 min), 54–70% B (12–12.1 min), 70–99% B (12.1–18 min), followed by re-equilibration to initial conditions. Mass spectrometry was performed in negative electrospray ionization mode over a mass range of m/z 50–1200. Source parameters were as follows: capillary voltage of −1.0 kV, cone voltage 30 V, source offset 80 V, source temperature 120 °C, and desolvation temperature 600 °C. Leucine-enkephalin (m/z 554.2615 [M − H]^−^) was used as a lock mass for real-time mass correction. Quality control (QC) samples were prepared by pooling equal aliquots of all samples and were injected at the beginning of the run and after every six injections to monitor instrument stability. The sample injection order was randomized to minimize analytical bias. Raw data were processed using Progenesis QI (version 2.0; Waters) for peak detection, alignment, and normalization. Lipid identification was performed by matching accurate mass, MS/MS fragmentation patterns, and retention times against public databases (HMDB, METLIN, LIPID MAPS) and an in-house library.

### Lipidomic analysis of cell-free culture media using LC-MS/MS

Lipidomic analysis of cell-free culture media was conducted to assess the lipid composition of the culture conditions: RPMI containing 10% FBS, RPMI containing 10% dFBS, and RPMI containing 1% dFBS supplemented with 1× B27-AO, and NIST SRM plasma (Sigma–Aldrich, NIST 1950) as a reference material. For lipid extraction, 200 μL of each medium sample was mixed with 800 μL of ice-cold methanol containing internal standards, including tridecanoic acid (0.4 μM, FFA 13:0, Sigma–Aldrich, 91988) and an 800-fold diluted SPLASH Lipidomix (Avanti Polar Lipids, 330707). The mixture was vortexed for 1 min and centrifuged at 18,500 × *g* for 10 min at 4 °C. A total of 600 μL of the supernatant was transferred to a new tube, followed by the addition of 360 μL of water and 770 μL of chloroform. After vigorous vortexing for 1 min and centrifugation, the lower organic phase was collected and dried under nitrogen gas. The lipids were reconstituted in 300 μL of isopropanol/acetonitrile/water (50:25:25, v/v/v) for LC-MS/MS analysis. Lipidomic analysis was conducted using the same LC-MS/MS conditions as described for intracellular lipid analysis.

### Isotope labeling and tracing analysis using LC-MS/MS

Stable isotope labeling in cells was accomplished by culturing A549 and H1299 cells in tracer medium supplemented with isotopic ^13^C_18_-LA (100 μM) for 4 h or 13.5 h. For *ELOVL5* knockout or FADS2 inhibitor experiments, cells were labeled under identical tracer conditions for 24 h. The intracellular lipids from A549 and H1299 were extracted in 400 μL of a 40:40:20 acetonitrile:methanol:water solution containing a 0.5% formic acid solution. The extracts were cleared by centrifugation. Tridecanoic acid (0.5 μM, FFA 13:0, Sigma-Aldrich, USA) and a 100-fold dilution of SPLASH Lipidomix (Avanti Polar Lipids, USA) were mixed with the extraction solution as internal standards. For *ELOVL5* knockout or FADS2 inhibitor-treated cells, lipids were extracted with 600 μL of ice-cold methanol: water (80:20, v/v) containing tridecanoic acid (0.08 μM, FFA 13:0, Sigma-Aldrich, USA) and an 8000-fold dilution of SPLASH Lipidomix (Avanti Polar Lipids, USA) as internal standard, using a scraper and transferred to microcentrifuge tubes. Samples were centrifuged at 18,500 x *g* for 15 min at 4 °C. A total of 500 μL of the supernatant was transferred to a new tube, followed by the addition of 800 μL of chloroform. The mixture was vortexed for 1 min and centrifuged for 15 min at 4 °C. The lower organic phase was collected, dried under a nitrogen stream, and reconstituted in 160 μL of isopropanol/acetonitrile/water (2:1:1, v/v/v). Intracellular lipids were analyzed using ultra-performance liquid chromatography-triple-quadrupole mass spectrometry (UPLC-TQ-MS) in multiple reaction monitoring (MRM) mode. An Agilent 1290 Infinity II LC coupled to an Agilent 6495 Triple Quadrupole MS system equipped with an Agilent Jet Stream ESI source (Agilent Technologies) at the Korea Basic Science Institute (KBSI, WS003) was used for the analysis of FFAs and PEs. Mass Hunter Workstation (ver. B.06.00, Agilent Technologies) software was used for data acquisition and analysis. Chromatographic separation of lipids in the cells was performed using an ACQUITY UPLC CSH C18 column (2.1 mm × 100 mm, 1.7 μm; Waters) at 30 °C and a flow rate of 0.2 mL/min. The binary mobile phases comprised 10 mM ammonium acetate in water:acetonitrile (60:40 v/v, solvent A) and isopropanol:acetonitrile (90:10 v/v, solvent B). Gradient elution was conducted as follows: 40–55% B from 0–6 min, 55–60% B from 6–11 min, 60–99% B from 11–14 min, 99% B from 14–18 min, 99–40% B from 18–18.1 min, and 40% B from 18.1–22 min. MS/MS experiments were conducted in negative ion mode. The MS system was operated using the following parameter settings: gas temperature of 220 °C, nebuliser gas of nitrogen at 30 psi, sheath gas temperature of 300 °C, and sheath gas flow rate of 11 L/min. The area was normalized to the protein.

### Cell culture

NCI-H1299 and A549 cells were purchased from the Korean Cell Line Bank (KCLB, Seoul, Korea). Cells were maintained in RPMI-1640 medium (Corning, 10-040- CVRC) supplemented with either 10% fetal bovine serum (FBS; Thermo, 10099141), 10% dialyzed FBS (dFBS; Gibco, 26400044), or 1X B27-plus supplement minus antioxidant (Thermo, 10889038) with 1% dFBS. All media were further supplemented with 1% antibiotic–antimycotic (Gibco, 15240062). Cells were incubated at 37 °C in a humidified atmosphere containing 5% CO₂.

### Generation and validation of the *ELOVL5*-KO A549 cell line

CRISPR/Cas9-mediated disruption of the ELOVL5 gene was performed using pSpCas9(BB)-2A-GFP (PX458; Addgene #48138) plasmid, as previously described [17]. Guide RNA (gRNA) targeting the human ELOVL5 gene (gRNA#1; 5′-GGCTGGGACCAAAATACATGAGG-3′) was cloned into pSpCas9(BB)-2A-GFP. A549 cells were transfected with constructs using Lipofectamine 3000 reagent (Invitrogen), and GFP-positive single cells were isolated by fluorescence-activated cell sorting (FACS) 24 h post-transfection. Single-cell clones were expanded, and *ELOVL5*-KO was confirmed by immunoblotting.

### Cell viability

Cell viability was determined by measuring the cellular ATP levels using CellTiter-Glo® 2.0 Assay (Promega, G9243) according to the manufacturer’s protocol. Cells were seeded at a density of 2 × 10⁴ cells/well in 200 µl of culture medium in 48-well plates. The following day, chemicals were added at the indicated concentrations and times. An equal volume of CellTiter-Glo® reagent was added directly to the medium, and the plates were incubated at room temperature for 10 min. Luminescence was measured using a Victor X2 microplate reader (PerkinElmer).

### Lipid peroxidation assay

Lipid peroxidation was assessed using C11-BODIPY 581/591 (BODIPY™ 581/591 C11, Invitrogen). Cells were treated with the indicated compounds for the times specified in the figure legends. After treatment, 2.5 µM C11-BODIPY was added, and cells were incubated at 37 °C for 15 min. Cells were detached with trypsin-EDTA, collected by centrifugation (300 × *g*, 3 min), and washed twice with cold DPBS. The cell pellets were resuspended in 200 µL of cold DPBS and kept on ice, protected from light until analysis. Flow cytometry was performed on a BD Accuri™ C6 Plus using a 488 nm excitation laser, and fluorescence was collected using the FITC (FL1) detector. At least 10,000 cells were analyzed per condition. Data acquisition and analysis were performed using BD Accuri C6 Plus software (BD Biosciences).

### Total RNA extraction and RT-qPCR

Total RNA was extracted using TRIzol™ Reagent (Thermo Fisher Scientific, 15596018; Invitrogen) according to the manufacturer’s instructions. First-strand complementary DNA (cDNA) was synthesized from 2 µg of total RNA using M-MLV reverse transcriptase (Promega, M1701). Quantitative RT–qPCR was performed with 24 ng of cDNA mixed with 2X Real-Time PCR Smart Mix containing EvaGreen fluorescent dye (Solgent, SRH71-M40H) on a CFX96™ Real-Time PCR Detection System (Bio-Rad Laboratories, USA).

The primer sequences used for qRT–PCR were as follows: FADS2 forward, 5′-TTAACTTCCAGATTGAGCACCA-3′ and reverse, 5′-TTGGCACATAGAGACTTCACC-3′; FADS1 forward, 5′-AGCCCAGCTGTGAGTTGCCCAAGAC-3′ and reverse, 5′-ATCGAGACAGGATGTGACTCCCTCC-3′; ELOVL5 forward, 5′-CATGTAACAAGGAGCTTCACCTTGTTTGAT-3′ and reverse, 5′-AATACTTTAATTTTGAATATCATACGAAAC-3′; ACSL4 forward, 5′-TCATGTGCTAGAACTGACAGC-3′ and reverse, 5′-GTACAGTCTCCTTTGCTTCCTT-3′; LPCAT3 forward, 5′-CACCGGCAACTACGATATCAA-3′ and reverse, 5′-CTTTCCCTCCGTCAAAGTAGT-3′; SCD1 forward, 5′-GCCAACACAATGGCATTCCAG-3′ and reverse, 5′-CCTCCTCTGGAACATCACCAG-3′; NRF2 forward, 5′-ACACGGTCCACAGCTCATC-3′ and reverse, 5′-TGTCAATCAAATCCATGTCCTG-3′; KEAP1 forward, 5′-ATTGGCTGTGTGTGGAGTTGC-3′ and reverse, 5′-CAGGTTGAACTCCTCTTG-3′; FSP1 forward, 5′-TCCGTCCGGCAGGAAGTGAA-3′ and reverse, 5′-CATTGAGAGGCAGCTCCTCC-3′; GPX4 forward, 5′-GCTGTGGAAGTGGATGAAGA-3′ and reverse, 5′-CAGCCGTTCTTGTCGATGA-3′; and β-Actin forward, 5′-CTGGCACCCAGCACAATG-3′ and reverse, 5′-GCCGATCCACACGGAGTACT-3′. All primers were synthesized by Cosmo Genetech (Korea).

### Western blotting

Cells were lysed in lysis buffer (50 mM Tris-HCl, pH 7.5; 150 mM NaCl; 0.5% NP-40; 0.5% Triton X-100; 0.1% sodium deoxycholate; and 1 mM EDTA) supplemented with a protease inhibitor cocktail. Protein lysates were separated by SDS–PAGE and transferred onto nitrocellulose membranes. Membranes were blocked with 5% skim milk in TBST (TBS with 0.1% Tween-20) for 1 h at room temperature, incubated with primary antibodies overnight at 4 °C, and then with HRP-conjugated secondary antibodies for 1 h at room temperature on a rocker. Blots were developed and analyzed using the FUSION Solo imaging system (Vilber Lourmat) and Evolution Capt software. For the detection of ELOVL5 protein, samples were not boiled before electrophoresis, as previously described.

The following primary antibodies were used: anti-FADS1 (Atlas Antibodies, HPA042705), anti-FADS2 (Invitrogen, PA5-87765), anti-ELOVL5 (Abcam, ab205535), anti-ACSL4 (Santa Cruz, sc-271800), anti-LPCAT3 (ProSci, 16-999), anti-NRF2 (Proteintech, 16396-1-AP), anti-KEAP1 (Proteintech, 10503-2-AP), anti-GPX4 (Abcam, ab125066), anti-FSP1 (Santa Cruz, sc-377120), anti-HSP90 (Santa Cruz, sc-13119), and anti-β-actin (Sigma-Aldrich, A5316).

### Statistical analyses

All experiments were independently repeated at least three times, and data are presented as mean ± standard deviation (SD). For experiments with small sample sizes (*n* = 3), statistical significance was assessed using non-parametric tests. Specifically, the Mann–Whitney U test was used for two-group comparisons, and the Kruskal–Wallis test followed by Dunn’s multiple-comparison test was applied for multiple group comparisons. Analyses were performed using GraphPad Prism 11 (GraphPad Software) and Microsoft Excel 2024 (Microsoft Office LTSC Professional Plus 2024). Significance was set at *p* < 0.05 (*), *p* < 0.01 (**), *p* < 0.001 (***), and *p* < 0.0001 (****).

## Supplementary information

Supplementary Figures

Supplementary Figure Legends

Full-length Uncropped Original Western Blots
