# Supplementary material for: Intrinsic polyunsaturated fatty acid synthesis capacity dictates ferroptosis sensitivity under restricted arachidonic acid availability
Source: Cell Death Discov. 2026 Jul 11;12:401. doi: 10.1038/s41420-026-03240-6 (PMC13639061; doi:10.1038/s41420-026-03240-6)

S1A

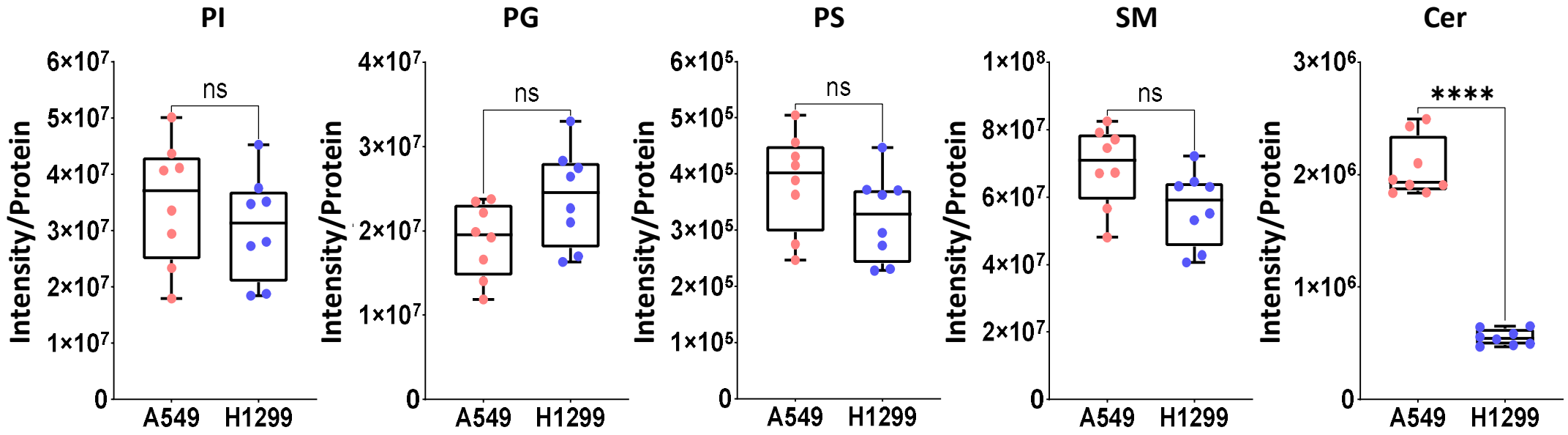
PI
PG
PS
SM
Cer
Intensity/Protein
ns
****
A549
H1299

S1B

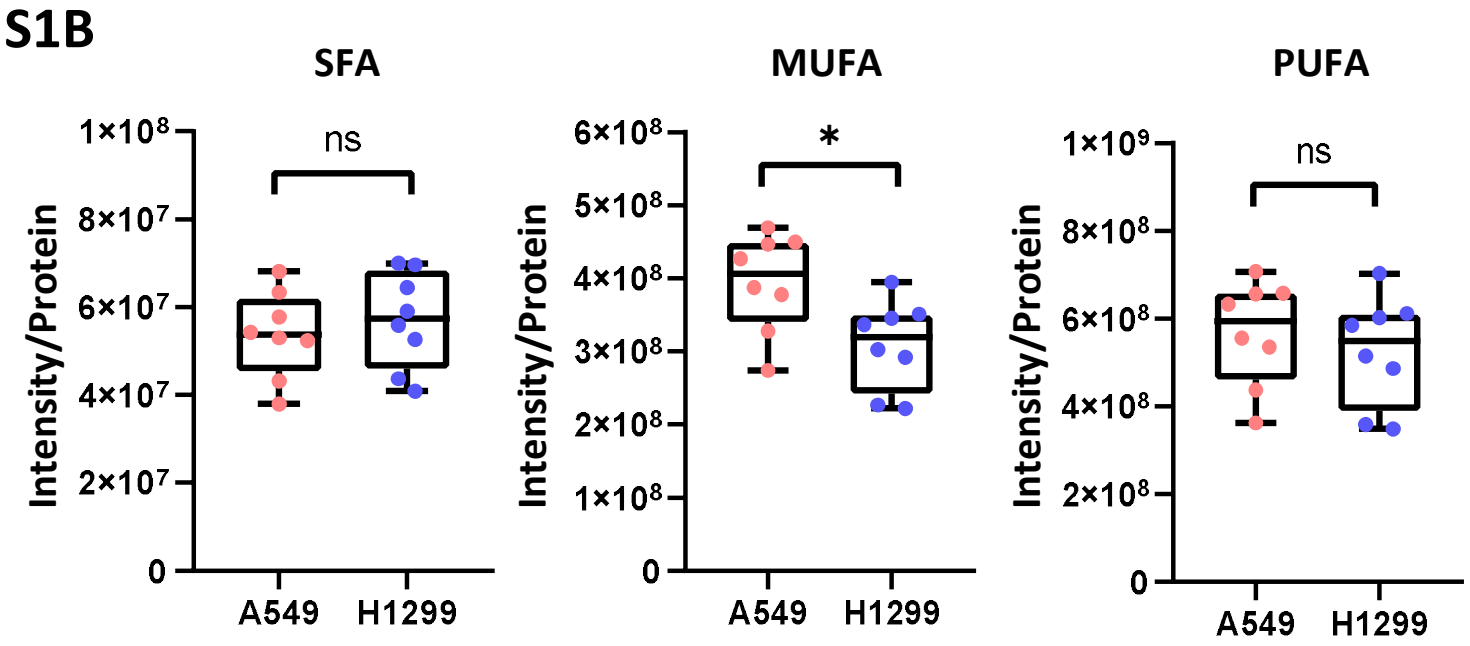
SFA
MUFA
PUFA
Intensity/Protein
ns
*
A549
H1299

S1C

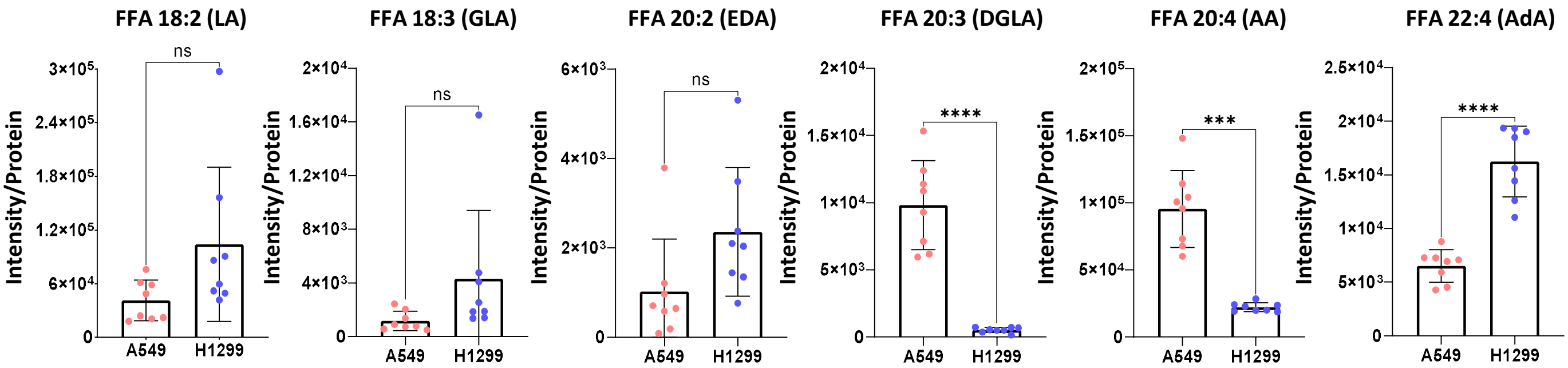
FFA 18:2 (LA)
FFA 18:3 (GLA)
FFA 20:2 (EDA)
FFA 20:3 (DGLA)
FFA 20:4 (AA)
FFA 22:4 (AdA)
Intensity/Protein
ns
****
***
A549
H1299

S2A

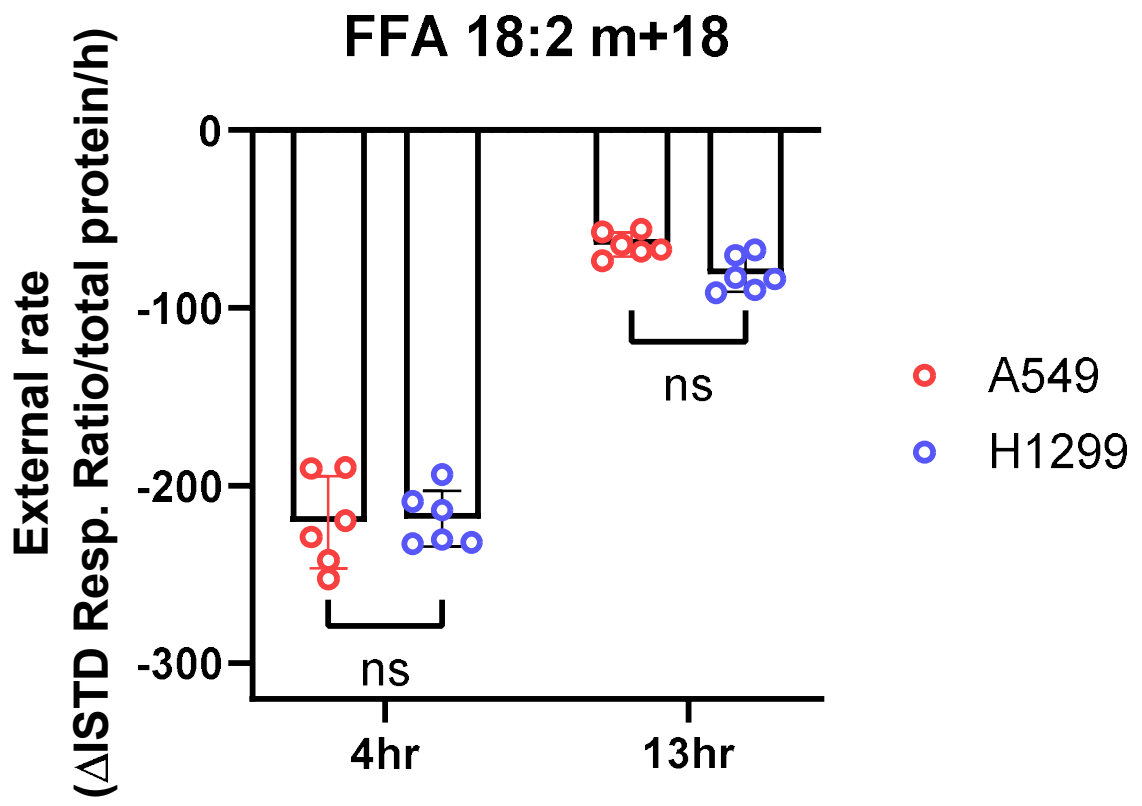
FFA 18:2 m+18
External rate
(ΔISTD Resp. Ratio/total protein/h)
0
-100
-200
-300
ns
ns
4hr
13hr
A549
H1299

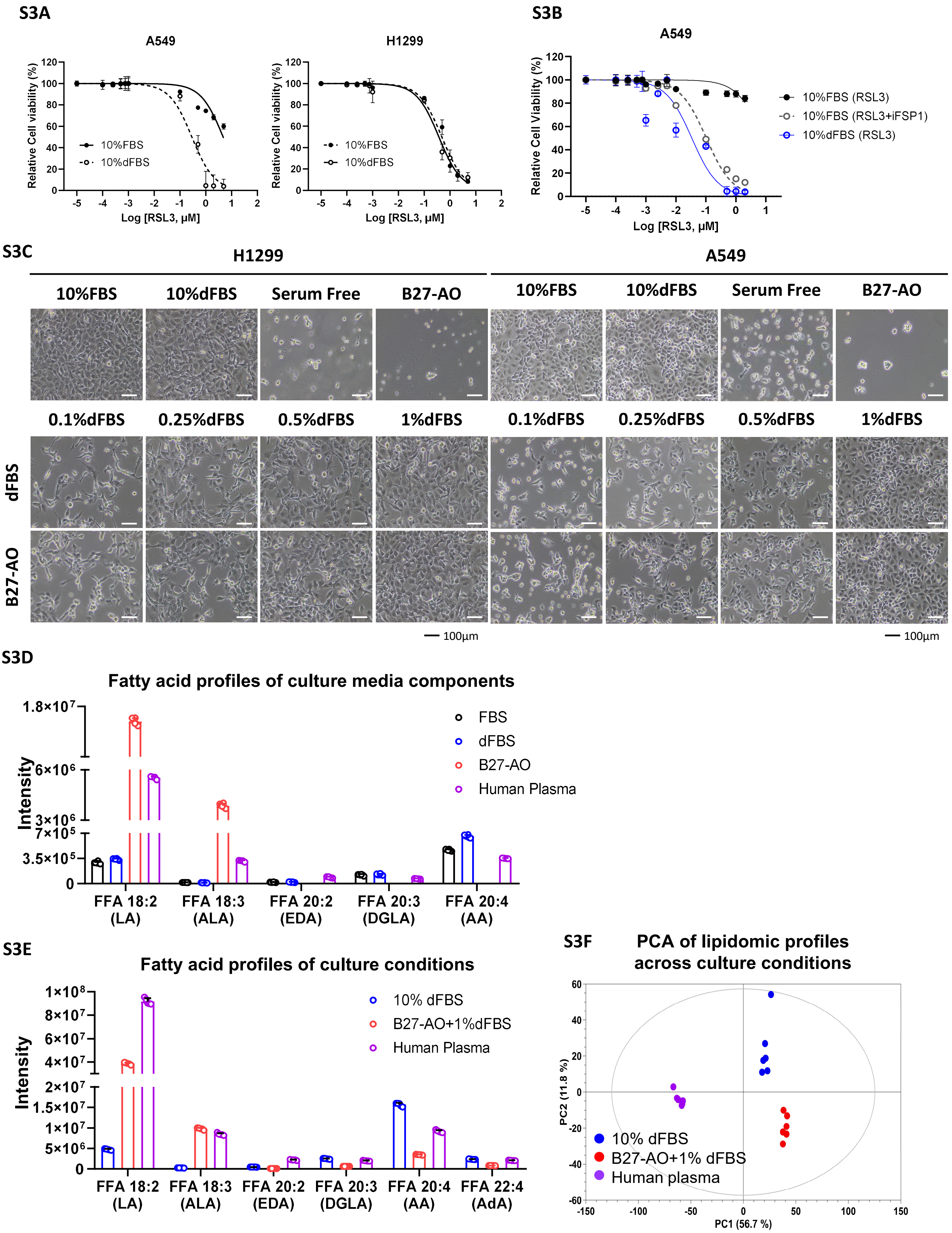
S3A
A549
Relative Cell viability (%)
120
100
80
60
40
20
0
10%FBS
10%dFBS
-5
-4
-3
-2
-1
0
1
2
Log [RSL3, μM]
H1299
Relative Cell viability (%)
10%FBS
10%dFBS
Log [RSL3, μM]
S3B
A549
Relative Cell Viability (%)
10%FBS (RSL3)
10%FBS (RSL3+iFSP1)
10%dFBS (RSL3)
Log [RSL3, μM]
S3C
H1299
A549
10%FBS
10%dFBS
Serum Free
B27-AO
0.1%dFBS
0.25%dFBS
0.5%dFBS
1%dFBS
dFBS
B27-AO
100μm
100μm
S3D
Fatty acid profiles of culture media components
Intensity
1.8×10^7
6×10^6
3×10^6
7×10^5
3.5×10^5
0
FBS
dFBS
B27-AO
Human Plasma
FFA 18:2 (LA)
FFA 18:3 (ALA)
FFA 20:2 (EDA)
FFA 20:3 (DGLA)
FFA 20:4 (AA)
S3E
Fatty acid profiles of culture conditions
Intensity
1×10^8
8×10^7
6×10^7
4×10^7
2×10^7
1.5×10^7
1×10^7
5×10^6
0
10% dFBS
B27-AO+1%dFBS
Human Plasma
FFA 18:2 (LA)
FFA 18:3 (ALA)
FFA 20:2 (EDA)
FFA 20:3 (DGLA)
FFA 20:4 (AA)
FFA 22:4 (AdA)
S3F
PCA of lipidomic profiles across culture conditions
PC2 (11.8 %)
60
40
20
0
-20
-40
-60
PC1 (56.7 %)
-150
-100
-50
0
50
100
150
10% dFBS
B27-AO+1% dFBS
Human plasma

S4A

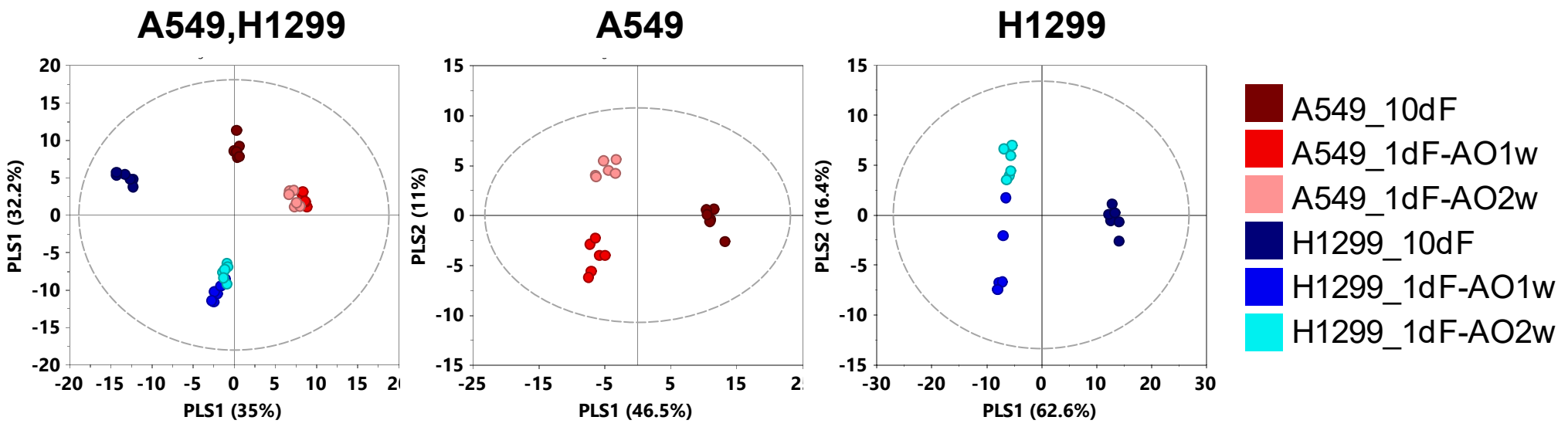
A549,H1299
A549
H1299
PLS1 (32.2%)
PLS1 (35%)
PLS2 (11%)
PLS1 (46.5%)
PLS2 (16.4%)
PLS1 (62.6%)
A549_10dF
A549_1dF-AO1w
A549_1dF-AO2w
H1299_10dF
H1299_1dF-AO1w
H1299_1dF-AO2w

S4B

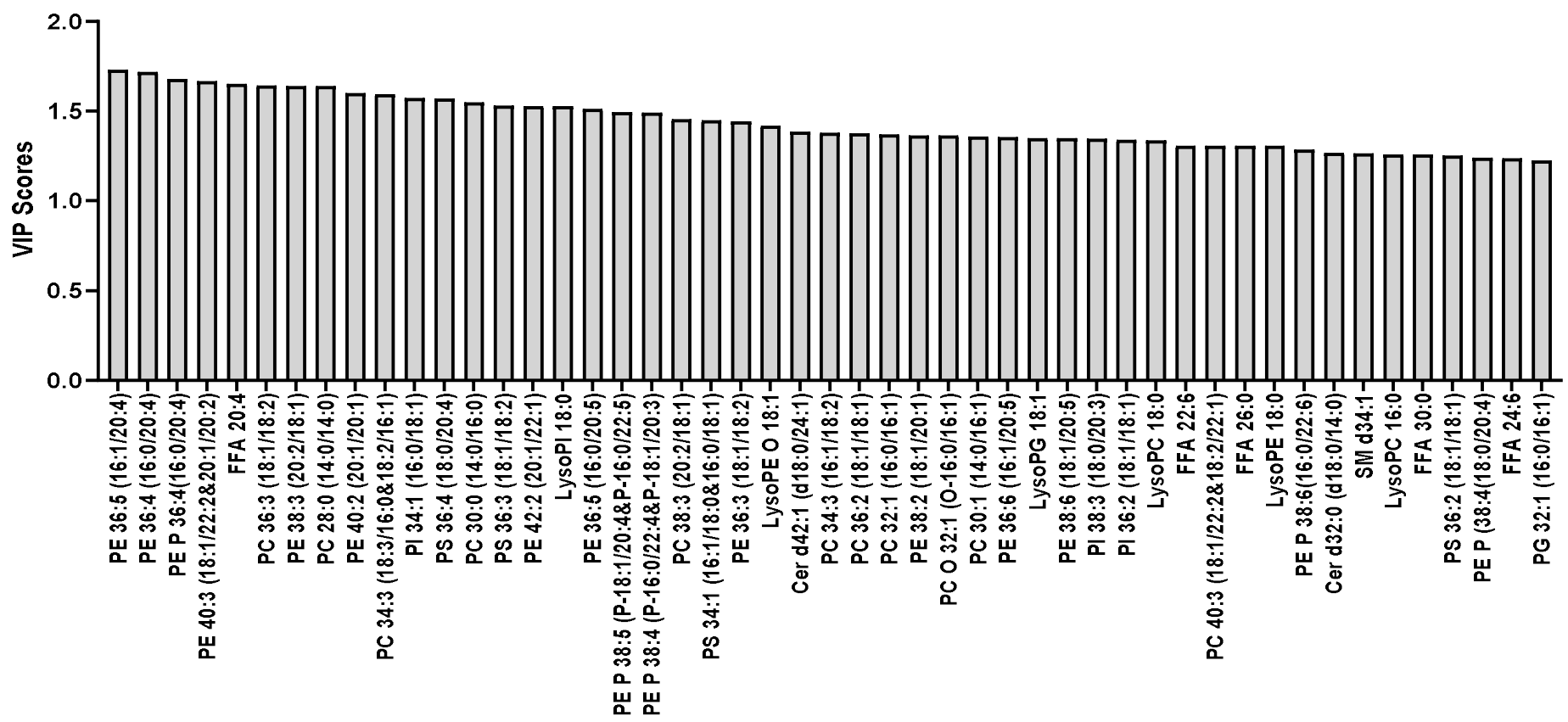
VIP Scores
PE 36:5 (16:1/20:4)
PE 36:4 (16:0/20:4)
PE P 36:4(16:0/20:4)
PE 40:3 (18:1/22:2&20:1/20:2)
FFA 20:4
PC 36:3 (18:1/18:2)
PE 38:3 (20:2/18:1)
PC 28:0 (14:0/14:0)
PE 40:2 (20:1/20:1)
PC 34:3 (18:3/16:0&18:2/16:1)
PI 34:1 (16:0/18:1)
PS 36:4 (18:0/20:4)
PC 30:0 (14:0/16:0)
PS 36:3 (18:1/18:2)
PE 42:2 (20:1/22:1)
LysoPI 18:0
PE 36:6 (16:0/20:6)
PE P 38:5 (P-18:1/20:4&P-16:0/22:6)
PE P 38:4 (P-16:0/22:4&P-18:0/20:3)
PC 38:3 (20:2/18:1)
PS 34:1 (16:1/18:0&16:0/18:1)
PE 36:3 (18:1/18:2)
LysoPE O 18:1
Cer d42:1 (d18:0/24:1)
PC 34:3 (16:1/18:2)
PC 36:2 (18:1/18:1)
PC 32:1 (16:0/16:1)
PE 38:2 (18:1/20:1)
PC O 32:1 (O-16:0/16:1)
PC 30:1 (14:0/16:1)
PE 36:6 (16:1/20:5)
LysoPG 18:1
PE 38:6 (18:1/20:5)
PI 38:3 (18:0/20:3)
PI 36:2 (18:1/18:1)
LysoPC 18:0
PC 40:3 (18:1/22:2&18:2/22:1)
FFA 22:6
FFA 26:0
LysoPE 18:0
PE P 38:6(16:0/22:6)
Cer d32:0 (d18:0/14:0)
SM d34:1
LysoPC 16:0
FFA 30:0
PS 36:2 (18:1/18:1)
PE P (38:4(18:0/20:4)
FFA 24:6
PG 32:1 (16:0/16:1)

S4C

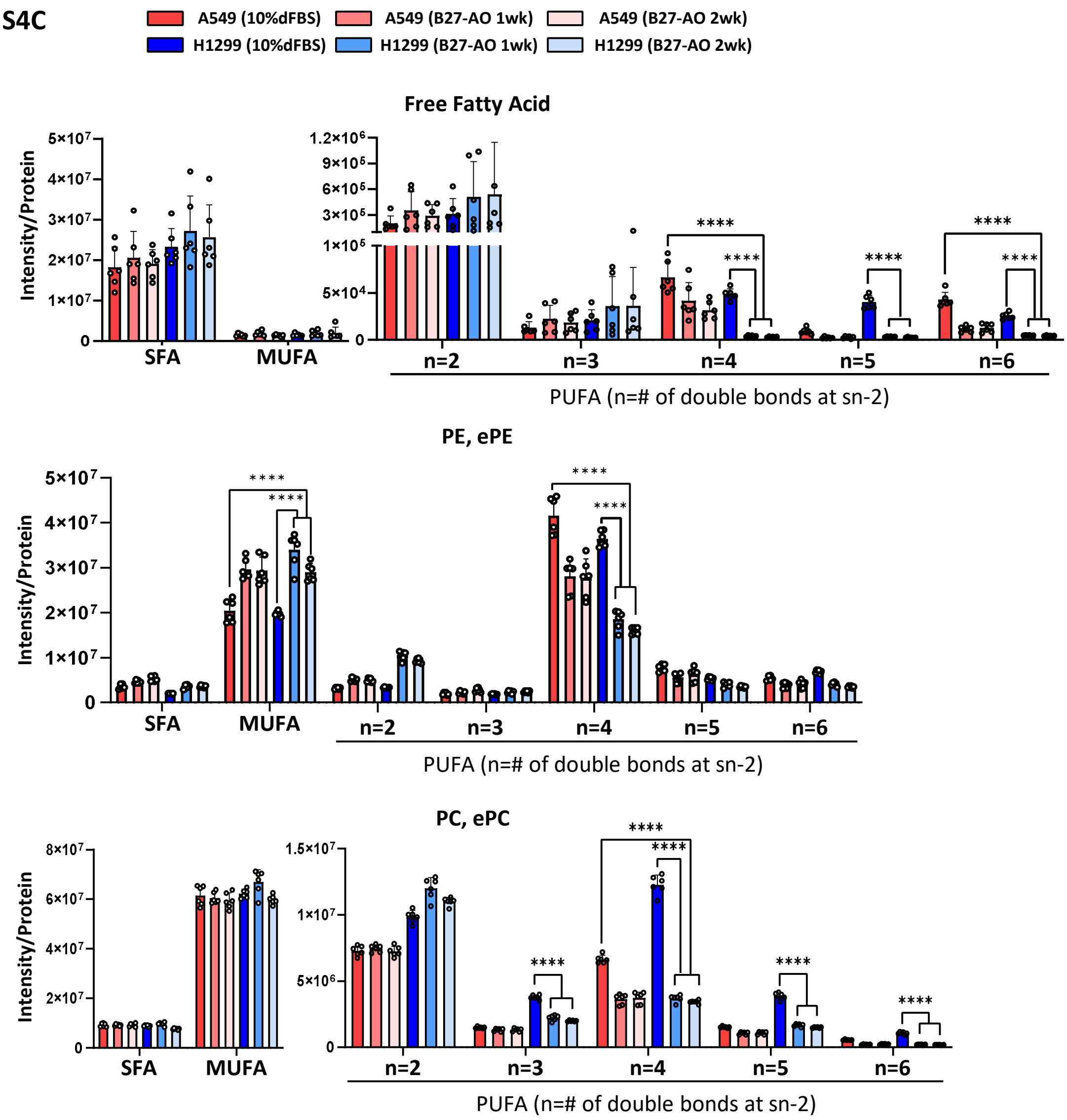
A549 (10%dFBS)
A549 (B27-AO 1wk)
A549 (B27-AO 2wk)
H1299 (10%dFBS)
H1299 (B27-AO 1wk)
H1299 (B27-AO 2wk)
Free Fatty Acid
Intensity/Protein
SFA
MUFA
n=2
n=3
n=4
n=5
n=6
PUFA (n=# of double bonds at sn-2)
PE, ePE
Intensity/Protein
SFA
MUFA
n=2
n=3
n=4
n=5
n=6
PUFA (n=# of double bonds at sn-2)
PC, ePC
Intensity/Protein
SFA
MUFA
n=2
n=3
n=4
n=5
n=6
PUFA (n=# of double bonds at sn-2)

S5A

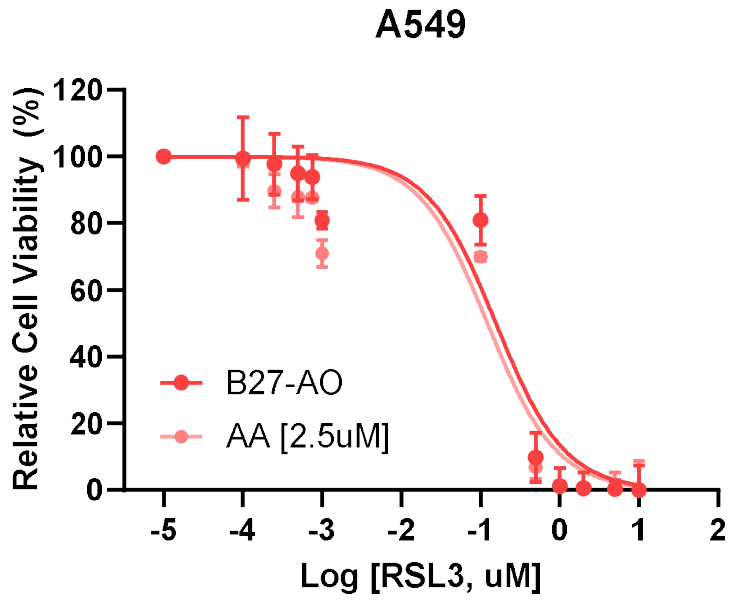
A549
Relative Cell Viability (%)
120
100
80
60
40
20
0
-5
-4
-3
-2
-1
0
1
2
Log [RSL3, uM]
B27-AO
AA [2.5uM]

S5B

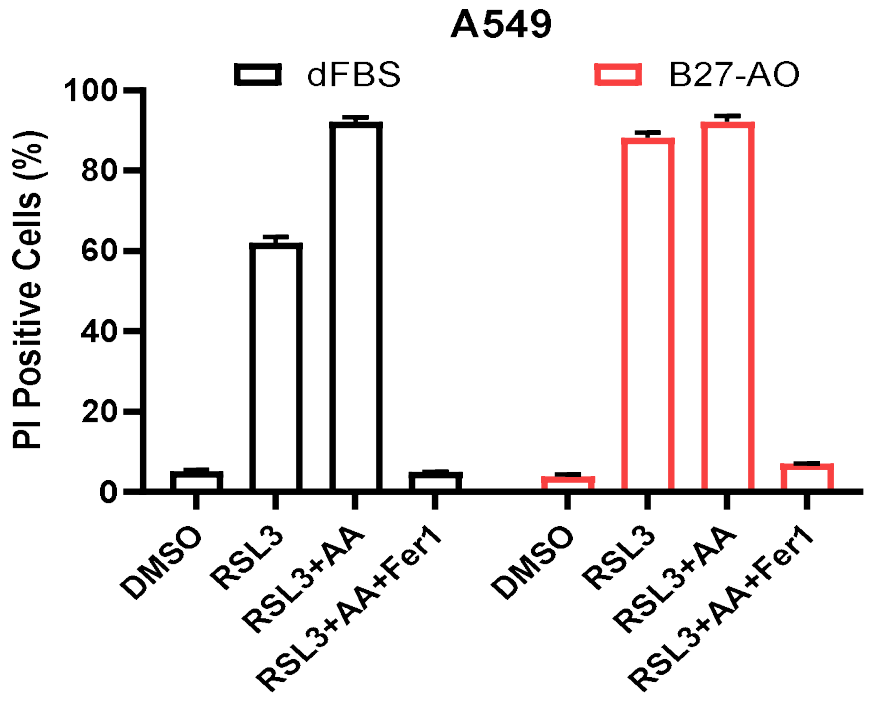
A549
dFBS
B27-AO
PI Positive Cells (%)
100
80
60
40
20
0
DMSO
RSL3
RSL3+AA
RSL3+AA+Fer1
DMSO
RSL3
RSL3+AA
RSL3+AA+Fer1

S5C

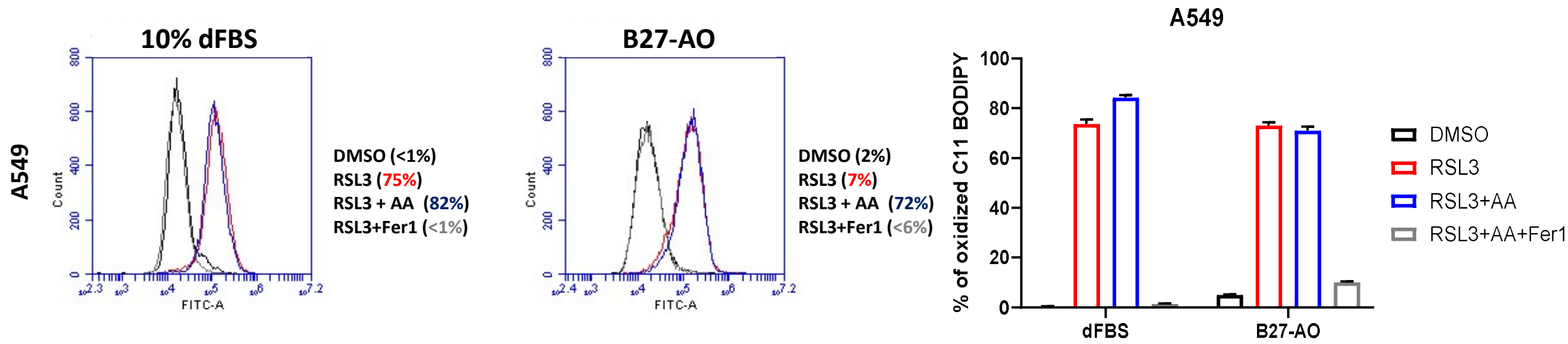
A549
10% dFBS
Count
FITC-A
DMSO (<1%)
RSL3 (75%)
RSL3 + AA (82%)
RSL3+Fer1 (<1%)
B27-AO
Count
FITC-A
DMSO (2%)
RSL3 (7%)
RSL3 + AA (72%)
RSL3+Fer1 (<6%)
A549
% of oxidized C11 BODIPY
100
80
60
40
20
0
dFBS
B27-AO
DMSO
RSL3
RSL3+AA
RSL3+AA+Fer1

S6 Whole membrane (Figure 2B)

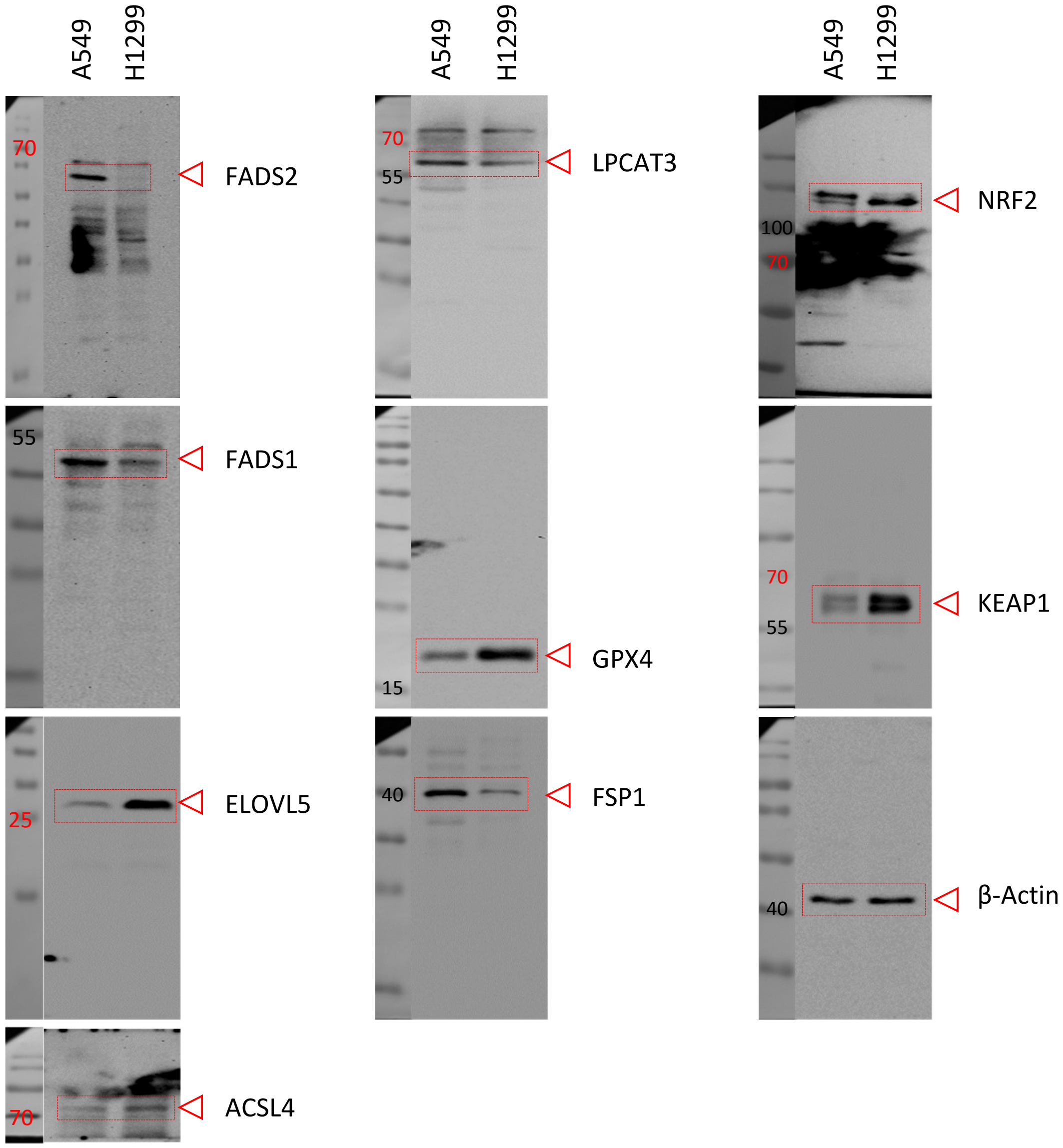

S7 **Whole membrane (Figure 3E)**

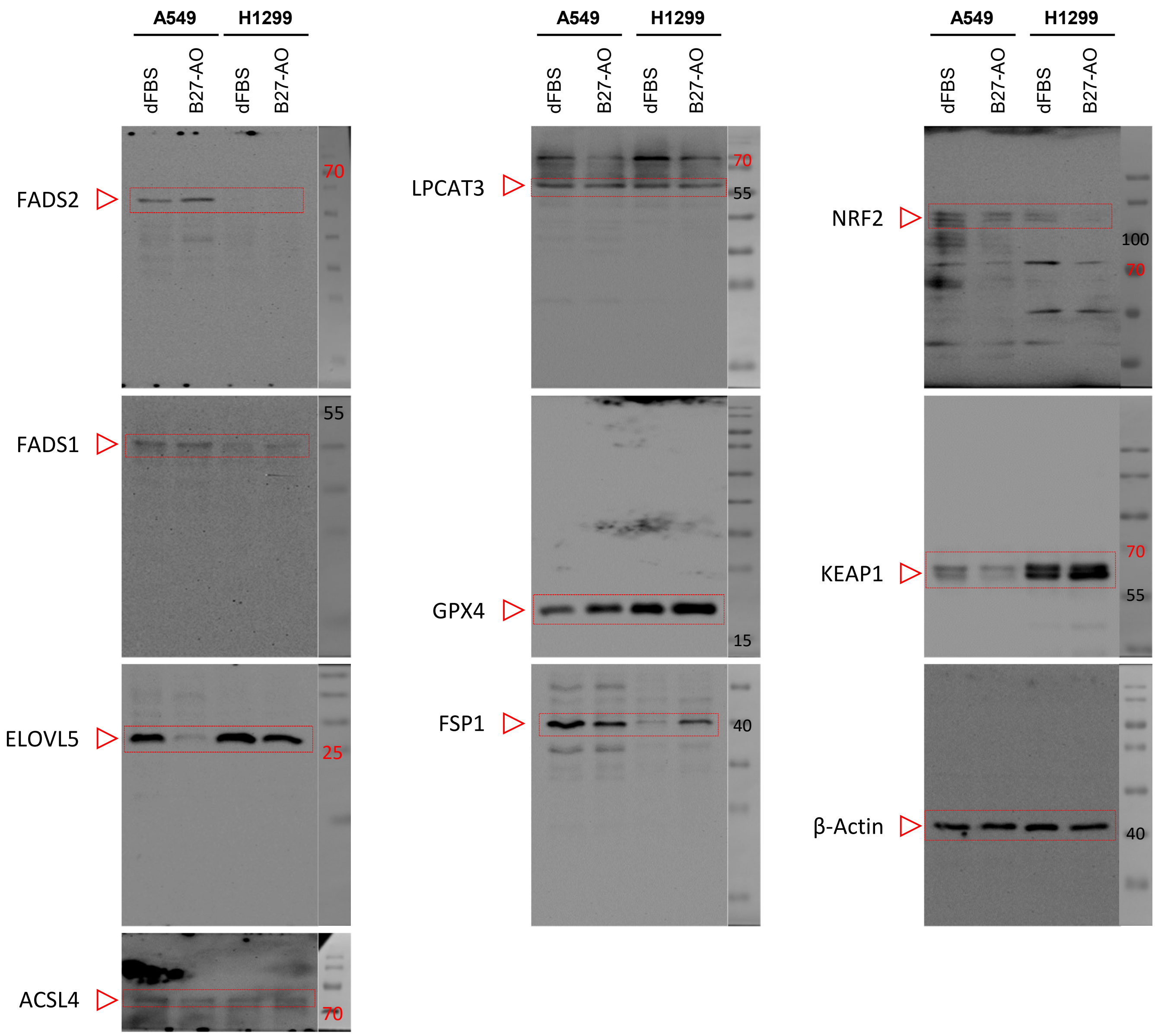

S8A

Whole membrane (Figure 3F)

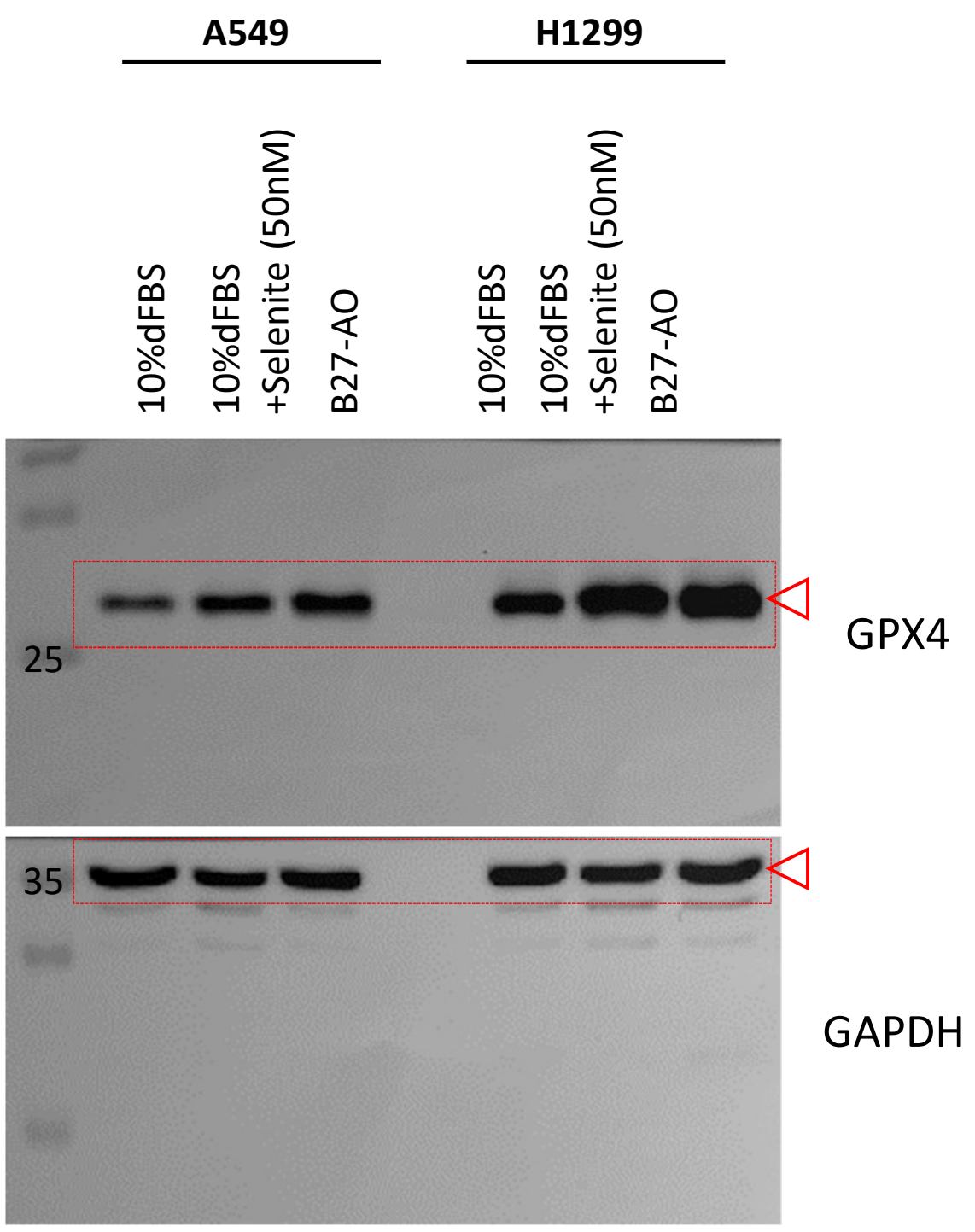

S8B

Whole membrane (Figure 5H)

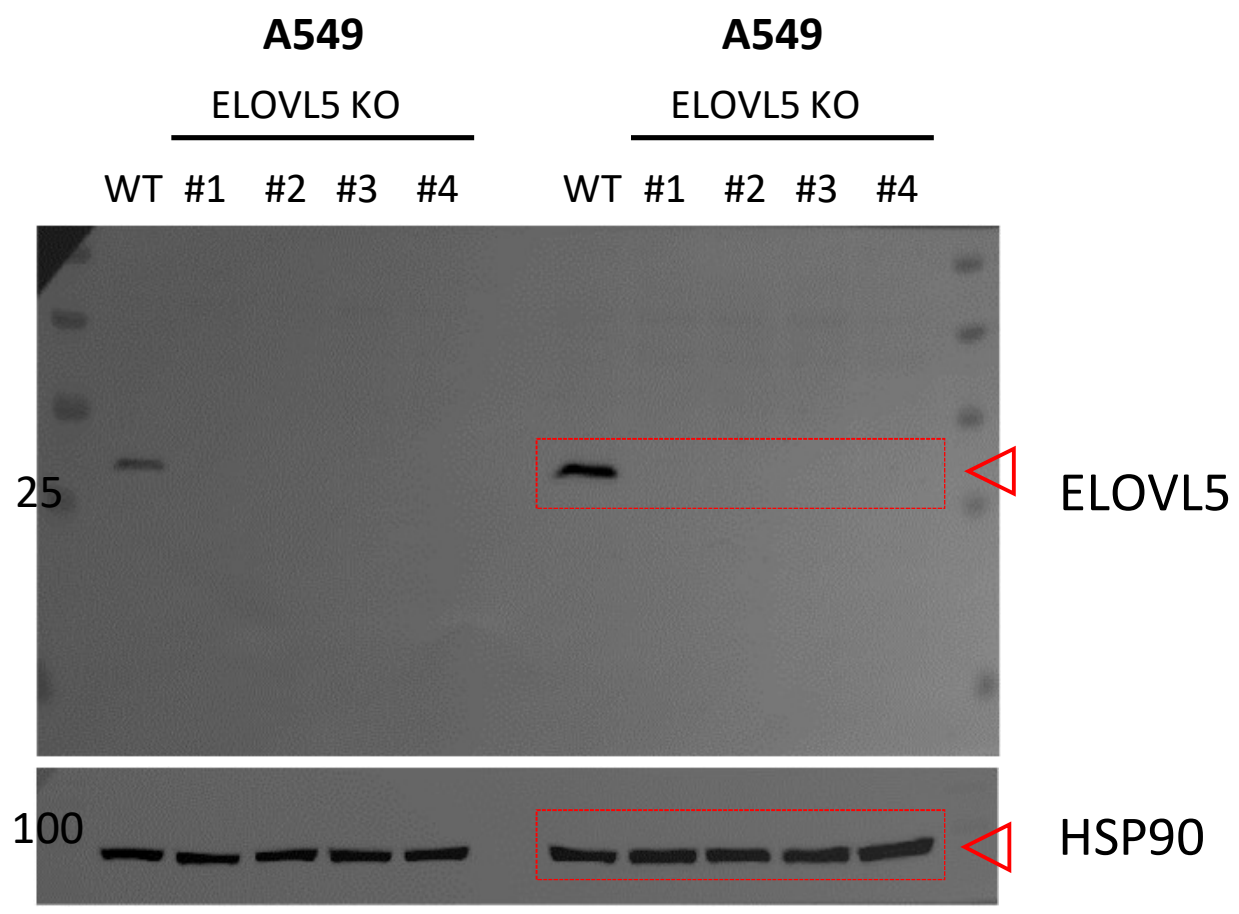

Supplement: Supplementary file 1 [file 41420_2026_3240_MOESM1_ESM.pdf]
