# Supplementary material for: Intrinsic polyunsaturated fatty acid synthesis capacity dictates ferroptosis sensitivity under restricted arachidonic acid availability
Source: Cell Death Discov. 2026 Jul 11;12:401. doi: 10.1038/s41420-026-03240-6 (PMC13639061; doi:10.1038/s41420-026-03240-6)

Corresponding figure: Figure 2B

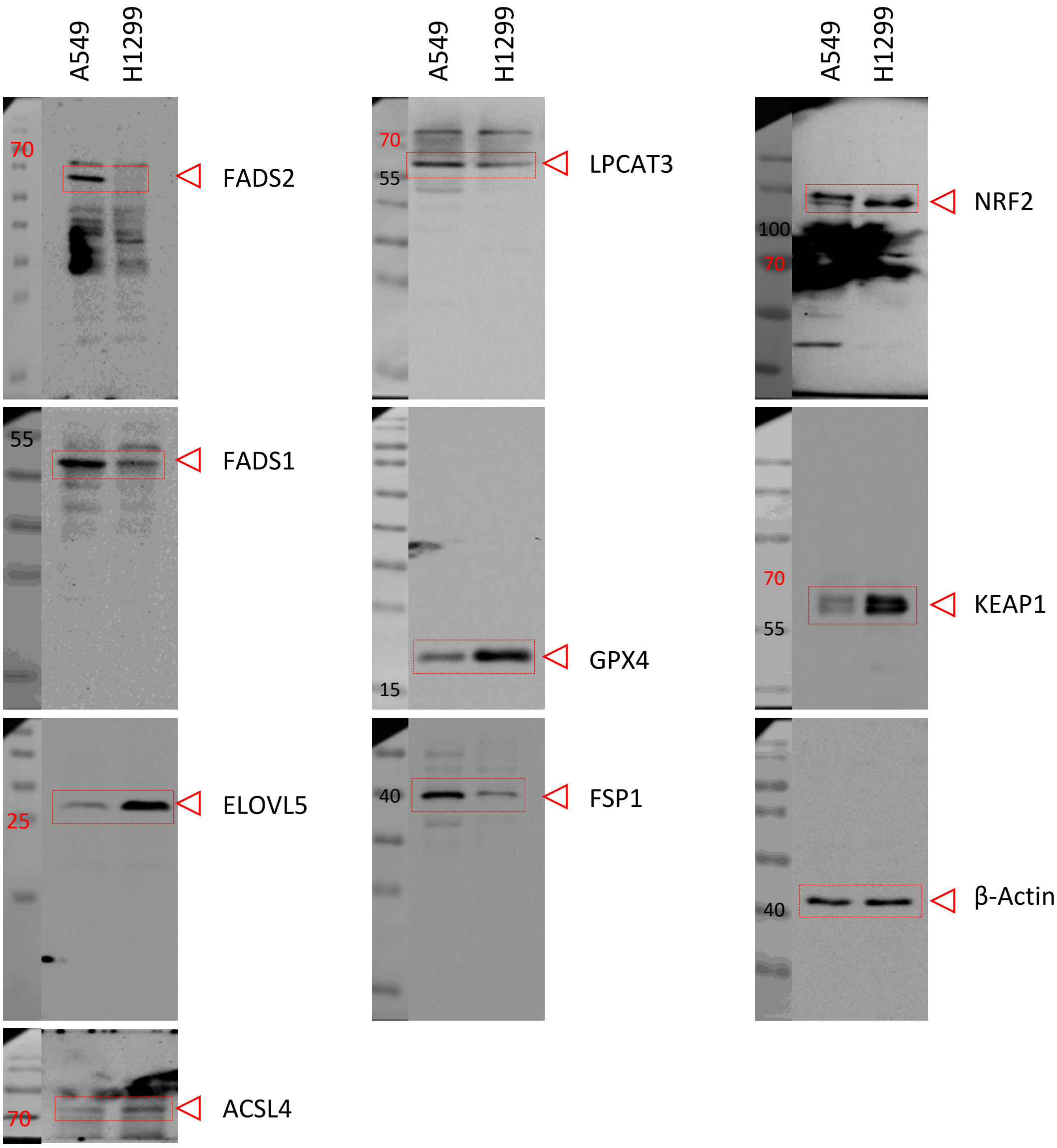

Corresponding figure: Figure 3E

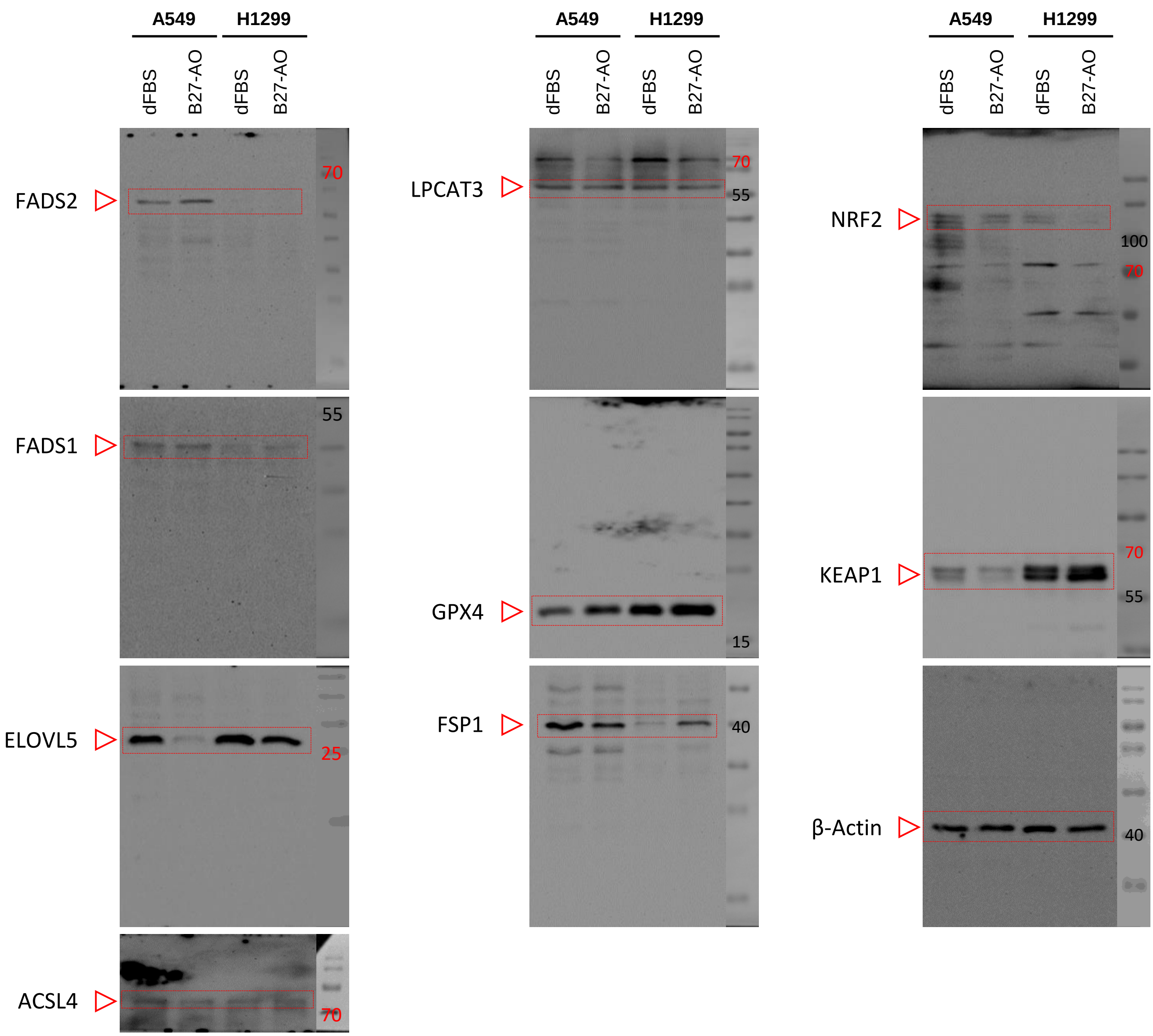

Corresponding figure: Figure 3F

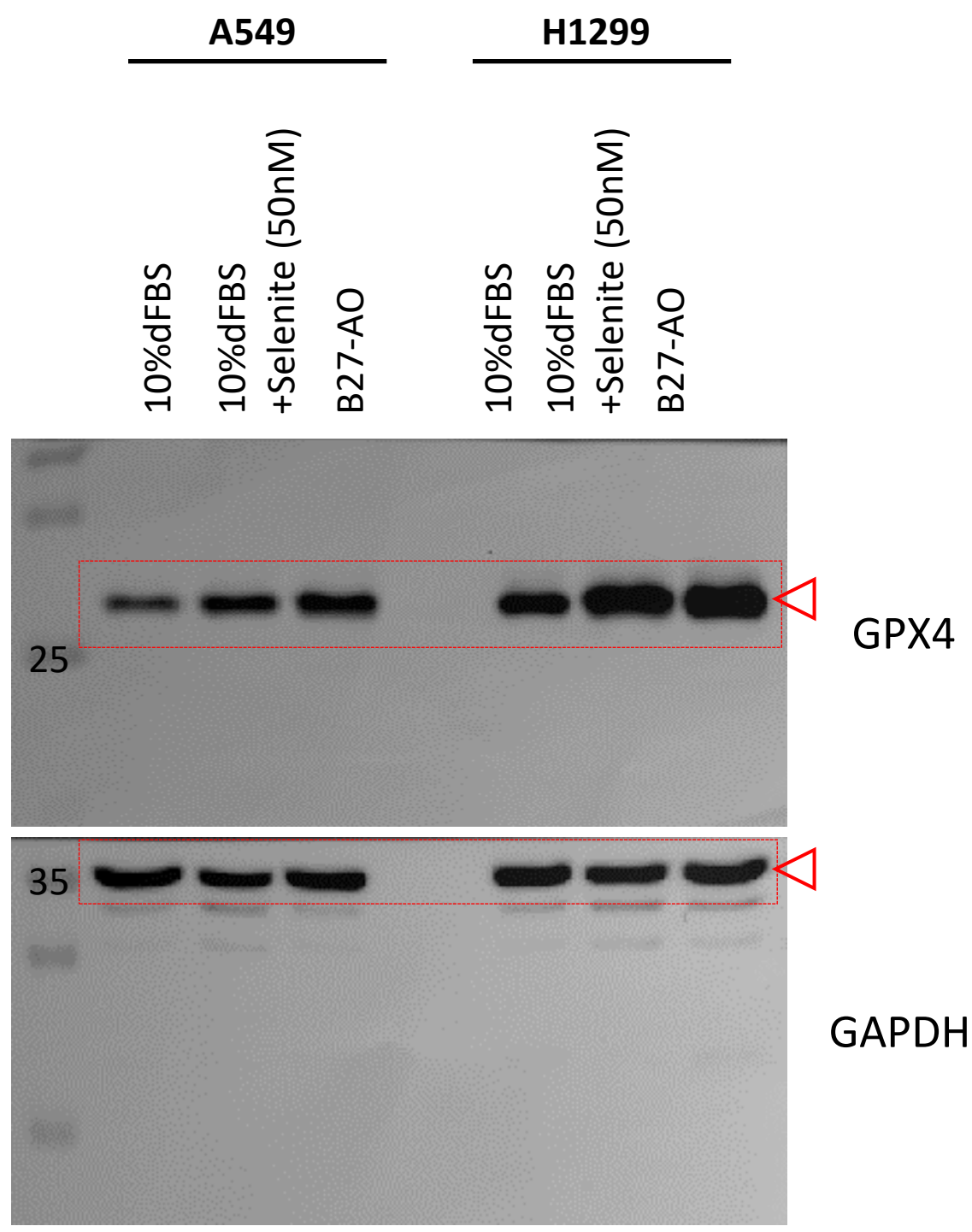

Corresponding figure: Figure 5H

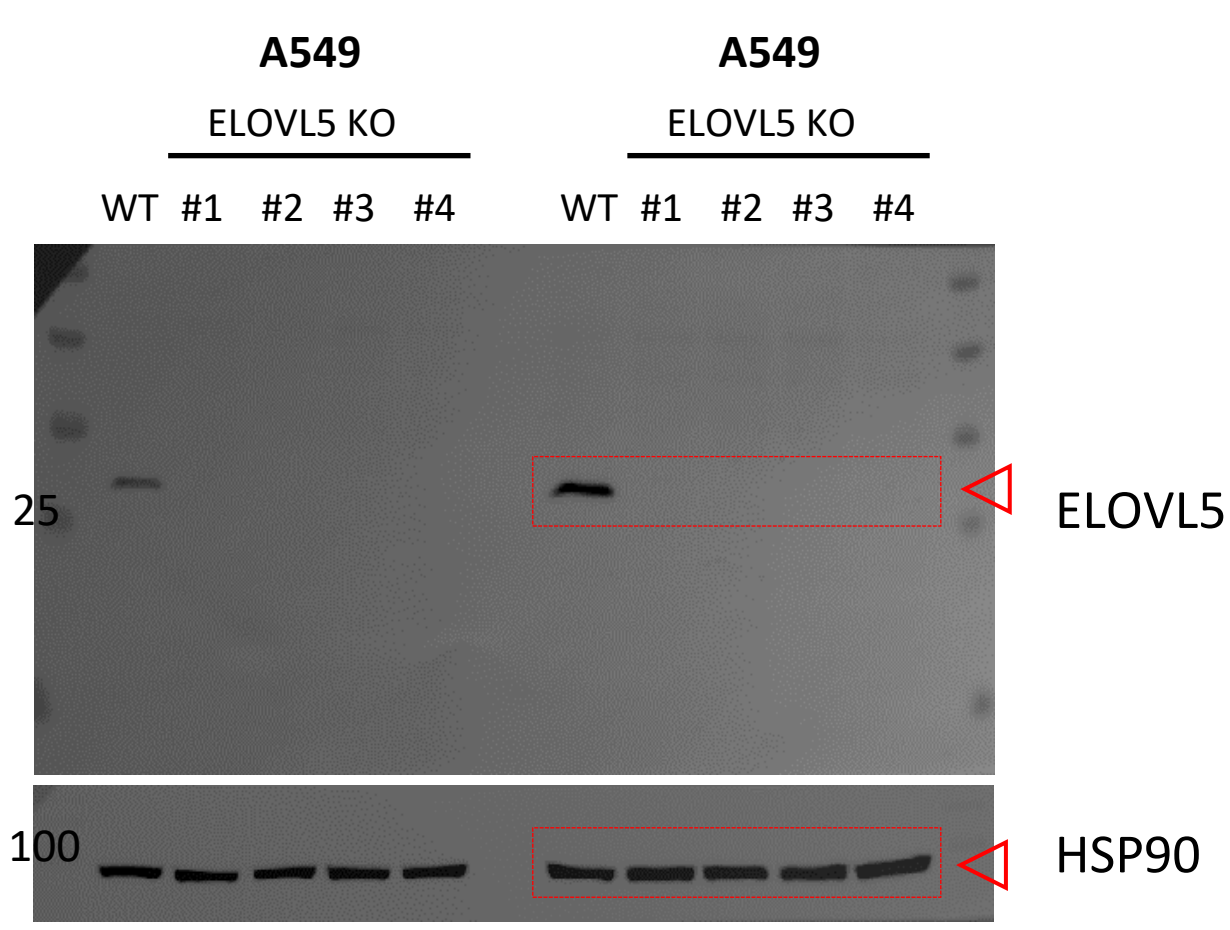

Supplement: Supplementary file 2 [file 41420_2026_3240_MOESM3_ESM.pdf]
