# Supplementary material for: Intrinsic polyunsaturated fatty acid synthesis capacity dictates ferroptosis sensitivity under restricted arachidonic acid availability
Source: Cell Death Discov. 2026 Jul 11;12:401. doi: 10.1038/s41420-026-03240-6 (PMC13639061; doi:10.1038/s41420-026-03240-6)
Supplement: Supplementary file 3 [file 41420_2026_3240_MOESM2_ESM.docx]

**Supplementary Figure Legends**

**Supplementary Figure S1. Expanded analysis of lipid subclasses and PUFA-related fatty acid composition in A549 and H1299 cells**

(S1A) Relative abundance of additional lipid subclasses, including phosphatidylinositol (PI), phosphatidylglycerol (PG), phosphatidylserine (PS), sphingomyelin (SM), and ceramides (Cer), normalized to protein (n = 8 independent experiments).
(S1B) Relative abundance of saturated fatty acids (SFA), monounsaturated fatty acids (MUFA), and polyunsaturated fatty acids (PUFA), normalized to protein (n = 8 independent experiments).
(S1C) Free fatty acid species involved in the PUFA biosynthetic pathway, including linoleic acid (LA), γ-linolenic acid (GLA), dihomo-γ-linolenic acid (DGLA), arachidonic acid (AA), and adrenic acid (AdA), normalized to protein levels (n = 8 independent experiments).
Data represent mean ± SD from n = 8 independent experiments. Statistical significance was determined by one-way ANOVA followed by Tukey’s multiple-comparison test (*P < 0.05; **P < 0.01; ***P < 0.001; ****P < 0.0001; ns, not significant).

**Supplementary Figure S2. Time-course analysis reveals comparable uptake of** **^13^C18-labeled linoleic acid in A549 and H1299 cells**

(S2A) Time-course analysis of ^13^C18-labeled linoleic acid in A549 and H1299 cells after 4 h and 13 h of labeling, normalized to protein. Data represent mean ± SD from n = 6 independent experiments. Statistical significance was determined using two-way ANOVA followed by Tukey’s multiple-comparison test (ns, not significant).

**Supplementary Figure S3. Ferroptosis responses and lipidomic characterization of A549 and H1299 cells under FBS, dialyzed FBS, and B27-AO culture conditions**

(S3A) Relative viability of A549 and H1299 cells cultured in 10% fetal bovine serum (FBS) or 10% dialyzed FBS (dFBS) and treated with RSL3 for 16 h (n = 3 independent experiments).
(S3B) Relative viability of A549 cells cultured in 10% FBS and treated with RSL3 with or without iFSP1 (3 μM), compared with A549 cells cultured in 10% dFBS and treated with RSL3 alone (n = 3 independent experiments).
(S3C) Representative morphology of A549 and H1299 cells 48 h after subculture in 10% FBS, 10% dFBS, serum-free medium, or B27-AO alone, as well as in B27-AO supplemented with 0.1%, 0.25%, 0.5%, or 1% dFBS. Scale bar, 100 μm.
(S3D) Fatty acid profiles of culture media components, including FBS, dFBS, B27-AO, and pooled human plasma standard reference material (SRM) (n = 6 for all conditions; human plasma, n = 4).
(S3E) Fatty acid profiles of culture conditions used in this study, including 10% dFBS and B27-AO supplemented with 1% dFBS (B27-AO conditions), compared with human plasma (n = 5).
(S3F) Principal component analysis (PCA) of lipidomic profiles across culture conditions (n = 5).
For panels A and B, data represent mean ± SD from n = 3 independent experiments. Statistical significance was determined using non-parametric tests: the Mann–Whitney U test for two-group comparisons and the Kruskal–Wallis test followed by Dunn’s multiple-comparison test for multiple-group comparisons, as appropriate (*P < 0.05; **P < 0.01; ***P < 0.001; ****P < 0.0001; ns, not significant).

**Supplementary Figure S4. Multivariate lipidomic analysis highlights PUFA-containing phospholipids as key determinants under lipid-restricted conditions.**

(S4A) Partial least squares discriminant analysis (PLS-DA) of global lipidomic profiles from A549 and H1299 cells cultured in dFBS or adapted to B27-AO/1% dFBS for 1 or 2 weeks (n = 6 independent experiments).
(S4B) Variable importance in projection (VIP) scores from the PLS-DA model, identifying lipid species that contribute most to separation across cell types and culture conditions.
(S4C) Relative abundance of free fatty acids (FFA), phosphatidylethanolamine and ether-linked PE (PE/ePE), and phosphatidylcholine and ether-linked PC (PC/ePC) species categorized by double-bond number, shown as intensity normalized to protein in A549 and H1299 cells cultured in 10% dFBS or adapted to B27-AO/1% dFBS for 1 or 2 weeks (n = 6 independent experiments).
For panel S4C, data represent mean ± SD from n = 6 independent experiments. Statistical significance was determined by one-way ANOVA followed by Tukey’s multiple-comparison test (*P < 0.05; **P < 0.01; ***P < 0.001; ****P < 0.0001; ns, not significant).

**Supplementary Figure S5. A549 cells remain ferroptosis-sensitive under dFBS and B27-AO conditions irrespective of arachidonic acid supplementation**

(S5A) Relative viability of A549 cells adapted to B27-AO/1% dFBS for 2 weeks, pretreated with arachidonic acid (AA, 2.5 μM) for 24 h, and subsequently treated with RSL3 for 20 h (n = 3 independent experiments).

(S5B) Propidium iodide (PI) uptake analysis by flow cytometry in A549 cells cultured in dFBS or B27-AO/1% dFBS and pretreated with AA (2.5 μM) for 24 h, followed by treatment with DMSO, RSL3 (1 μM), or RSL3 in combination with ferrostatin-1 (Fer-1, 2 μM) for 20 h (n = 3 independent experiments).

(S5C) Lipid peroxidation analysis by C11-BODIPY staining in A549 cells cultured in dFBS or B27-AO/1% dFBS and pretreated with AA (2.5 μM) for 24 h, followed by treatment with DMSO, RSL3 (1 μM), or RSL3 in combination with ferrostatin-1 (Fer-1, 2 μM). Left, representative histograms; right, quantification of lipid peroxidation measured 1 h after RSL3 treatment (n = 3 independent experiments).

Data represent mean ± SD from n = 3 independent experiments. Statistical significance was determined using non-parametric tests: the Mann–Whitney U test for two-group comparisons and the Kruskal–Wallis test followed by Dunn’s multiple-comparison test for multiple-group comparisons, as appropriate (*P < 0.05; **P < 0.01; ***P < 0.001; ****P < 0.0001; ns, not significant).

**Supplementary Figure S6. Uncropped whole-membrane immunoblots corresponding to Figures 2B**

Full, uncropped western blot images corresponding to the immunoblot data presented in Figure 2B are shown. Membranes were processed and imaged under the same experimental conditions as in the main figures. Dashed lines indicate the regions shown in the corresponding main figure, and red arrowheads indicate the bands of interest. Molecular weight markers (kDa) are indicated on the left.

**Supplementary Figure S7. Uncropped whole-membrane immunoblots corresponding to Figure 3E**

Full, uncropped western blot images corresponding to the immunoblot data presented in Figure 3E are shown. Membranes were processed and imaged under the same experimental conditions as in the main figures. Dashed lines indicate the regions shown in the corresponding main figure, and red arrowheads indicate the bands of interest. Molecular weight markers (kDa) are indicated on the right.

**Supplementary Figure S8. Uncropped whole-membrane immunoblots corresponding to Figures 3F and 5H**

Full, uncropped western blot images corresponding to the immunoblot data presented in Figure 3F (A) and Figure 5H (B) are shown. All membranes were processed and imaged under the same experimental conditions as in the main figures, red arrowheads indicate the bands of interest, and molecular weight markers (kDa) are indicated on the left.
